# Mechanism of action of *sprG1*-encoded type I toxins in *Staphylococcus aureus*: from membrane alterations to mesosome-like structures formation and bacterial lysis

**DOI:** 10.3389/fmicb.2023.1275849

**Published:** 2023-10-03

**Authors:** Laurence Fermon, Agnès Burel, Emeline Ostyn, Stéphane Dréano, Arnaud Bondon, Soizic Chevance, Marie-Laure Pinel-Marie

**Affiliations:** ^1^Univ Rennes, INSERM, BRM – UMR_S 1230, Rennes, France; ^2^Univ Rennes, CNRS, ISCR – UMR 6226, Rennes, France; ^3^Univ Rennes, CNRS, INSERM, BIOSIT – UAR 3480, US_S 018, Rennes, France; ^4^Univ Rennes, CNRS, INSERM, IGDR – UMR 6290, Rennes, France

**Keywords:** type l toxin-antitoxin systems, *Staphylococcus aureus*, membrane depolarization, ATP depletion, membrane permeabilization, mesosome-like structures

## Abstract

*sprG1/*SprF1 is a type I toxin-antitoxin system located on *Staphylococcus aureus* prophage. It has previously been shown that the two toxins, SprG1_31_ and SprG1_44_, encoded by the *sprG1* gene, are two membrane-associated peptides structured in a single α-helix. Overexpression of these two peptides leads to growth inhibition and even *S. aureus* death. In this study, we investigated the involvement of each peptide in this toxicity, the sequence requirements necessary for SprG1_31_ toxicity, and the mechanism of action of these two peptides. Our findings show that both peptides, when expressed individually, are able to stop growth, with higher toxicity observed for SprG1_31_. The combination of a hydrophobic domain and a charged domain located only at the C-terminus is necessary for this toxicity, likely to retain the orientation of the transmembrane domain. A net cationic charge for SprG1_31_ is not essential to induce a growth defect in *S. aureus*. Furthermore, we established a chronology of toxic events following overexpression to gain insights into the mode of action of SprG1_44_ and SprG1_31_. We demonstrated that mesosome-like structures are already formed when membrane is depolarized, about 20 min after peptides induction. This membrane depolarization occurs concomitantly with a depletion of intracellular ATP, leading to *S. aureus* growth arrest. Moreover, we hypothesized that SprG1_44_ and SprG1_31_ do not form large pores in the *S. aureus* membrane, as ATP is not excreted into the extracellular medium, and membrane permeabilization is delayed relative to membrane depolarization. The next challenge is to identify the conditions under which SprG1_44_ and SprG1_31_ are naturally expressed, and to uncover their potential roles during staphylococcal growth, colonization, and infection.

## Introduction

Toxin-antitoxin (TA) systems are widespread two-component genetic modules in bacterial genomes. They encode a stable toxic protein, whose ectopic overexpression can cause growth arrest or cell death, and an unstable antitoxin, which inhibits the toxin’s activity during bacterial growth. They are classified into eight types depending on the antitoxin nature and its mode of action. While the toxins are mainly proteins (except for type VIII TA systems where toxins are RNAs), the antitoxins can be either non-coding RNAs (in type I, III, and VIII systems) or small proteins (in types II, IV, V, VI, and VII) (Jurėnas et al., 2022). A high antitoxin-toxin ratio has no impact on bacterial growth, while its decrease leaves toxins free to disrupt essential cellular functions such as DNA replication, protein synthesis, or cell division (Goeders and Van Melderen, 2014). Initially discovered in plasmids where they ensure plasmid maintenance through post-segregational killing (PSK), the biological functions attributed to chromosomal TA systems are currently the maintenance of mobile genetic elements and the regulation of bacterial physiology and fitness (Jurėnas et al., 2022).

In type I TA systems, antitoxins are antisense RNAs that interact with toxin-encoding mRNAs by pairing, thereby inhibiting toxin mRNA translation and/or promoting its degradation. Under stressful conditions, the RNA antitoxin pool is reduced by specific RNases, which in turn induces toxin translation. Type I toxins are classified into two categories: membrane-associated type I toxins and cytosolic type I toxins (Brielle et al., 2016). The membrane-associated type I toxins generally contain less than 60 amino acids, are hydrophobic and have a putative α-helical transmembrane domain, similar to phage holins (Saier and Reddy, 2015) or cationic antimicrobial peptides (Huang et al., 2010). Thus, when overexpressed, some of them are able to induce morphological changes in bacteria, membrane depolarization and/or permeabilization, followed by intracellular ATP depletion. We recently described the current state of knowledge on the mechanisms of action of some membrane-associated type I toxins by establishing a chronology of their toxic effects on the bacterial cell (Nonin-Lecomte et al., 2021).

The *Staphylococcus aureus* human pathogen expresses functional type I TA systems (Pinel-Marie et al., 2014; Habib et al., 2018; Germain-Amiot et al., 2019; Riffaud et al., 2019). *S. aureus* is a serious multidrug-resistant pathogen and a leading cause of bacteremia and infective endocarditis as well as osteoarticular, skin and soft tissue, pleuropulmonary, and device-related infections (Tong et al., 2015). We have recently characterized the *sprG1*/SprF1 TA systems located within the φN315 prophage in *S. aureus* (Pinel-Marie et al., 2014, 2021). The transcription of the *sprG1* gene generates two transcripts differing at their 5′-ends, the *sprG1_312_* predominant form and the *sprG1_439_* minor form. *SprG1* mRNAs encode two membrane peptides from a single internal reading frame: a long (44 amino acids, SprG1_44_ also named PepG1_44_) and a short version (31 amino acids, SprG1_31_ also named PepG1_31_). These two peptides are hydrophobic and have an α-helical transmembrane domain solved by NMR (Nonin-Lecomte et al., 2021). The SprG1_44_ peptide has 13 extra amino acids in the N-terminus compared to SprG1_31_. Ectopic overexpression of both *sprG1*-encoded peptides induces *S. aureus* cell death due to a disruption of membrane integrity within 1 h upon induction. The dual-function RNA antitoxin SprF1 promotes *sprG1* mRNA degradation and prevents *sprG1* mRNA translation by interacting in *cis* with its overlapping 3′-end (Pinel-Marie et al., 2014). Moreover, thanks to a purine-rich sequence located at its 5′-end, SprF1 also interacts with ribosomes, which could promote translation attenuation and persister cell formation (Pinel-Marie et al., 2021). We have shown that extracellular addition of chemically-synthesized peptides SprG1_44_ and SprG1_31_ or of membrane extracts prepared from *S. aureus* cells overexpressing *sprG1*-encoded peptides triggers the lysis of both competing bacteria (Gram-negative and positive bacteria) and human erythrocytes (Pinel-Marie et al., 2014). However, the precise mechanism of action of these peptides on bacterial and mammalian membranes remains unclear.

In this work, we investigated the mechanism of action of the two membrane-associated type I toxins, SprG1_44_ and SprG1_31_. We focused on the perturbations induced by the two peptides on the membrane of *S. aureus* under overexpression conditions. We showed that these peptides induced membrane depolarization and a depletion in intracellular ATP, leading to membrane permeabilization without large pores formation. Moreover, the appearance of mesosome-like structures, concomitant with membrane depolarization, was observed when these peptides were overexpressed. We also demonstrated by a mutagenesis approach that the toxicity of SprG1_31_ is due to the combination of its α-helical transmembrane domain and charged amino acids positioned at the C-terminal domain, presumably to maintain the orientation of the α-helix.

## Materials and methods

### Strains and plasmid constructions

Bacterial strains, plasmids, and primers used in this work are listed in Supplementary Tables S1–S3, respectively. To generate an anhydrotetracycline (aTc)-inducible construct for *sprG1_312_* expressing the two *sprG1_312_*-encoded peptides (SprG1_31_ and SprG1_44_), a DNA fragment containing the *sprG1_312_* sequence starting at +1 nt of transcription and ending at its 3′-end was amplified from N315 genomic DNA by PCR using KOD Hot Start polymerase (Novagen) (Table 1). For the aTc-inducible construct for *sprG1_312_* expressing SprF1 antitoxin, the *sprG1_312_* sequence corresponds to a 374-bp DNA fragment starting at +1 nt of transcription and ending 63 nts downstream from its 3′-end. These two constructs were used to generate the following mutants. To generate an inducible construct containing a ‘*sprG1_312_*-STOP_1_’ mutant expressing only SprG1_31_, we constructed a premature TAA termination codon by replacing A54 with a thymine, and T55 and G56 with two adenines (Supplementary Figures S1, S2; Table 1). For the “*sprG1_312_*-M14A” mutant expressing only SprG1_44_, the ATG93 initiation codon was replaced by an GCC alanine codon (Supplementary Figures S1, S2; Table 1). For the “*sprG1_312_*-STOP_1,2,14_” and “*sprG1_312_*-STOP_1,2,14,17_” mutants not expressing SprG1_44_ and SprG1_31_, the three or four codons (ATG54, GTG57, ATG93, and ATT102) were replaced by a TAA termination codon (Supplementary Figures S1, S2). For the flagged *sprG1_312_* construct, a DNA sequence encoding for the 1xFLAG or 3xFLAG epitopes was added downstream the ATG93 initiation codon or upstream from the termination codon, in-frame with the *sprG1_312_*-encoded peptides. For the ‘SprG1_31_Δ9’ and “SprG1_31_Δ2” mutants, we generated a truncation of 9 amino acid residues or 2 amino acid residues, respectively, at the C-terminal part of SprG1_31_ (Table 2). For the “SprG1_31_-K2K3” mutant, the C-terminal two lysin residues was placed at the N-terminal part of SprG1_31_ (Table 2). For the “SprG1_31_-F10A-F13A” and “SprG1_31_-F10E-F13E” mutants, the two phenylalanine residues at position 10 and 13 were replaced by alanine residues or glutamic acid residues, respectively (Table 2). All the PCR products were digested with KpnI and EcoRI restrictions enzymes (NEB Biolabs) and ligated using T4 DNA ligase (NEB Biolabs) into the corresponding sites of pALC plasmid (Bateman et al., 2001). All plasmid constructs were confirmed by Sanger sequencing using 3130xl Genetic Analyzer capillary electrophoresis laser-coupled system (ABI PRISM). Plasmids were then transformed into *Escherichia coli* XL1 blue chemically competent cells, into shuttle *S. aureus* RN4220 strain (Kreiswirth et al., 1983) and, finally, into *S. aureus* N315 strain deleted for endogenous *sprG1*/*sprF1* locus (Pinel-Marie et al., 2014).

**Table 1:** Sequences of *sprG1_312_*-encoded peptides overexpressed in *S. aureus.*

Plasmids	Peptide(s) overexpressed	Peptide sequence	Construction without the SprF1 antitoxin?
pALCΩ*sprG1_312_*	SprG1_44_ + SprG1_31_	MVALLKSLERRRLMITISTMLQFGLFLIALIGLVIKLIELSNKK	No
		MITISTMLQFGLFLIALIGLVIKLIELSNKK	
pALCΩ*sprG1_312_-STOP_1_*	SprG1_31_	MITISTMLQFGLFLIALIGLVIKLIELSNKK	No
pALCΩ*sprG1_312_-M14A*	SprG1_44_	MVALLKSLERRRLAITISTMLQFGLFLIALIGLVIKLIELSNKK	Yes

**Table 2:** Amino acid sequence, net charge, hydrophobicity of wild-type and mutants of *sprG1_312_*-encoded peptides.

	Amino acid sequence^a^	Net charge^b^	Hydrophobicity^b^
SprG1_44_	MVALLKSLERRRLMITISTMLQFGLFLIALIGLVIKLIELSNKK	4.98	1.01
SprG1_31_	MITISTMLQFGLFLIALIGLVIKLIELSNKK	1.98	1.39
SprG1_31_∆9	MITISTMLQFGLFLIALIGLVI	−0.02	2.29
SprG1_31_∆2	MITISTMLQFGLFLIALIGLVIKLIELSN	−0.02	1.75
SprG1_31_-K2K3	MKKITISTMLQFGLFLIALIGLVIKLIELSN	1.98	1.39
SprG1_31_-F10A-F13A	MITISTMLQ**A**GL**A**LIALIGLVIKLIELSNKK	1.98	1.32
SprG1_31__F10E-F13E	MITISTMLQ**E**GL**E**LIALIGLVIKLIELSNKK	−0.02	0.98
SprG1_31_-1xFLAG-Nt	MDYKDDDDKTISTMLQFGLFLIALIGLVIKLIELSNKK	−1.02	0.42
SprG1_31_-1xFLAG-Ct	MITISTMLQFGLFLIALIGLVIKLIELSNKKDYKDDDDK	−1.02	0.42
SprG1_31_-3xFLAG-Ct	MITISTMLQFGLFLIALIGLVIKLIELSNKKDYKDDDDKDYKDDDDKDYKDDDDK	−7.01	−0.67

a Hydrophobic amino acids are shown in red, negatively charged amino acids in green and positively charged amino acids in blue. Mutations are underlined.

b The charge and the hydrophobicity index (based on Kyte-Doolittle scale) have been calculated thanks to the R package «Peptides» (Osorio et al., 2015).

### *S. aureus* growth conditions, *sprG1_312_* induction and growth kinetics

*S. aureus* strains containing the relevant plasmids were grown overnight in Mueller Hinton broth (MH, Oxoid) with 10 μg/mL chloramphenicol. The cultures were then diluted to an optical density at 600 nm (OD_600_) of 0.05 in MH and grown at 37°C with shaking for approximately 2.5 h until the exponential growth phase in 96-well microplates (OD_600_ ≈ 0.15) or in 50 mL tubes (OD_600_ ≈ 0.4). To induce the expression of *sprG1_312_*, the cultures were then incubated with aTc, initially diluted in 100% ethanol, at a final concentration of 0.25 μM. An equivalent concentration of ethanol was added for the vehicle control. For the growth kinetics, the OD_600_ was measured at 30-min intervals using a Synergy 2 microplate reader (Bio Tek).

### RNA extractions and northern blots

Thirty minutes after induction, bacteria were centrifuged for 10 min at 4,500 rpm at 4°C, and the pellets were stored at −80°C. Cell pellets were resuspended in 500 μL lysis buffer (0.5% SDS, 20 mM sodium acetate, 1 mM EDTA pH 5.5) and mechanically broken through beads beating in phenol pH 4 using a FastPrep-24 5G instrument (MP Biomedicals). After 5 min centrifugation at 13,000 rpm and 4°C, the aqueous phase was transferred with an equal volume of phenol (pH 4) and centrifuged 5 min at 13,000 rpm and 4°C. This was then transferred with an equal volume of a 24:1 solution of chloroform/isoamyl alcohol, and centrifuged for 5 min at 13,000 rpm and 4°C. RNAs were precipitated from the aqueous phase by adding 2.5 volumes of ethanol and 0.1 volume of 3 M NaOAc (pH 5.2) solution. For the northern blot assays, 10 μg of RNAs were separated onto an 8% urea-PAGE gel, and electro-transferred onto a ZetaProbe GT membrane (Bio-Rad) in 0.5x TBE buffer (90 mM Tris, 90 mM boric acid, 2 mM EDTA) for 2 h at 25 V. RNAs were cross-linked to the membrane by UV irradiation. Specific ^32^P probes (see Supplementary Table S3) were labeled with 0.5 μL [γ^32^P]-ATP (5 μCi) and T4 PNK enzyme, and hybridized overnight on the membranes using ExpressHyb solution (Ozyme). Membranes were washed twice in 2x SSC (0.3 M sodium chloride, 0.03 M sodium citrate, pH 7.0) solution with 0.05% SDS for 10 min, in 0.1x SSC with 0.1% SDS for 10 min, then exposed and scanned with a PhosphorImager.

### Protein extractions, cell fractionation and western blots

Forty-five minutes after aTc induction, 2 mL cultures from strains carrying the empty pALC vector and constructions with flagged peptides, were centrifuged at 13,000 rpm for 1 min. The pellets were resuspended in lysis buffer (50 mM Tris-EDTA, pH 7.7, 5 mM MgCl_2_, and 100 μg/mL lysostaphin), incubated for 15 min at 37°C, and transferred onto ice with protease inhibitors (Roche Diagnostics) added to a final concentration of 1x. After sonication, the proteins were quantified using the Qubit Protein Assay Kit (ThermoFisher), as per the manufacturer’s instructions. For cell fractionation, the pellets were resuspended into 50 mM Tris–HCl, pH 7.5, 20 mM MgCl_2_, supplemented with 30% sucrose and lysostaphin (0.1 mg/mL). The suspension was incubated 10 min at room temperature and centrifuged at 4,000 rpm at 4°C for 8 min. The pellet was dissolved into 50 mM Tris–HCl, pH 7.5, 20 mM MgCl_2_ with protease inhibitors (Roche Diagnostics) added to a 1x final concentration and mechanically broken through beads beating using a FastPrep-24 5G instrument (MP Biomedicals). After 60 min centrifugation at 19,000 rpm and 4°C, the supernatant contained the cytoplasmic protein fraction, while the pellet included the membrane protein fraction. The membrane fraction was then dissolved in 50 mM Tris–HCl, pH 7.5, 20 mM MgCl_2_, 3 μg/mL Triton-X100. For the western blots, 2.5 μg of proteins were loaded and separated onto a 4–16% Tricine SDS-PAGE gel. Gels were run at 50 V for 30 min and then at 120 V for 90 min. Gels were electro-transferred onto Amersham Hybond-P PVDF membranes (GE Healthcare) at 30 V, 4°C for 2 h. After blocking, membranes were incubated with monoclonal mouse anti-FLAG M2 antibody (1:2000, Sigma-Aldrich). Incubation was carried out for 1 h at room temperature, and the membranes were washed and then revealed with an Amersham ECL Plus Western Blotting Detection kit, and scanned with an ImageQuant LAS 4000 imager (GE Healthcare). Coomassie blue staining was done as a loading control.

### Measurement of *S. aureus* membrane depolarization and permeabilization

For the fluorescence-based microplate assays, 2 mL cultures from *S. aureus* strains grown until exponential growth phase were centrifuged at 5,000 g for 5 min. Bacteria were resuspended in PBS supplemented with 25 mM glucose, to place the bacterial cell in a high-energy state (Clementi et al., 2014), and incubated at 37°C under agitation for 15 min. 5 μM SYTOX Green (Invitrogen) or 0.5 μg/mL DiBAC_4_(3) (Sigma Aldrich) were added, respectively, before or after the incubation of 15 min at 37°C. Bacterial cultures (100 μL) were loaded in a black 96-well microplate. DiBAC_4_(3) and SYTOX green fluorescence was measured with excitation and emission wavelengths of 488 nm and 525 nm, respectively, using the Synergy 2 microplate reader with continuous shaking during 10 min at 37°C, sufficient time to stabilize fluorescence. Then, 0.25 μM aTc, 0.01% ethanol used as vehicle control or 12.5 μg/mL nisin or 28 μg/mL vancomycin used as the positive controls were added to the corresponding wells. OD_600_ and fluorescence were then measured every 2 min for 100 min at 37°C with continuous shaking. For the time-point measurements, *S. aureus* strains grown until exponential growth phase in MH and incubated with 0.25 μM aTc or 0.1% ethanol at 37°C with shaking at 160 rpm. Then, at each time point, 0.5–1 mL of cultures were centrifuged at 5,000 g for 3 min. Bacteria were resuspended in PBS and stained with 0.5 μg/mL DiBAC_4_(3) by incubation at 37°C for 4 min in the dark or 2.5 μM SYTOX Green by incubation at 37°C with continuous shaking for 15 min. Then, OD_600_ and fluorescence were measured using the Synergy 2 microplate reader. Fluorescence values were normalized with OD_600_.

### Measurement of intracellular and extracellular ATP

*S. aureus* strains were grown at 37°C until exponential growth phase in MH and incubated with 0.25 μM aTc or 0.1% ethanol at 37°C with shaking at 160 rpm. For each time point, 0.75 mL cultures were centrifuged at 5,000 g for 3 min. Supernatants or bacteria resuspended in MH were incubated at 37°C with BacTiter-Glo Microbial Cell Viability Assay (Promega) at an equal volume in a black 96-well microplate. After a 4 min incubation in the dark, OD_600_ and luminescence were measured using the Synergy 2 microplate reader. Luminescence values were normalized with OD_600_.

### Transmission electron microscopy

*S. aureus* strains were grown until exponential growth phase and then incubated with 0.25 μM aTc or 0.1% ethanol for 20 min and 50 min at 37°C with shaking at 160 rpm. Cultures were centrifuged at 2,500 g for 5 min. Cell pellets were fixed for 24 h with 2.5% glutaraldehyde in 0.1 M cacodylate buffer (pH 7.2), rinsed with the same buffer, post-fixed for 2 h with 1% osmium tetroxide and rinced. Samples were embedded in 3.5% low melting agar and then dehydrated in a graded series of increasing ethanol concentrations (50, 70, 90, and 100% v/v). Samples were infiltrated with a mixture of ethanol-epon resin (50/50) for 3 h. Infiltration was continued the next day with 2,4,6-Tris dimethylaminomethyl phenol-30 (DMP30)-epoxy resin for 3 h. Samples were finally embedded in a new mix of DMP30-epoxy resin and polymerized at 60°C for 24 h. From the resin-embedded samples blocks, ultrathin sections (80 nm) were then cut with a UCT Leica ultramicrotome, placed on grids, poststained for 30 min with uranyl acetate and viewed with a Jeol 1,400 TEM (Jeol Ltd) supplied with a Gatan Orius camera (Gatan Inc). Images were taken from two different experiments for each condition, and four different areas were investigated under each condition.

### Statistical information

The data are presented as mean ± standard deviation (SD) calculated using Prism 9 software (GraphPad Software). A *p* value <0.05 was considered the threshold for statistical significance. Statistical analysis was performed using the unpaired two-tailed Student’s *t*-test or the two-way ANOVA with Tukey’s correction with 95% confidence intervals (CI) in GraphPad Prism 9. *p* value significance intervals (*) and the number of biological replicates (*n* value) are indicated within each figure legend.

## Results

### SprG1_31_ and SprG1_44_, individually or together, trigger *S. aureus* growth inhibition

**Figure 1:**
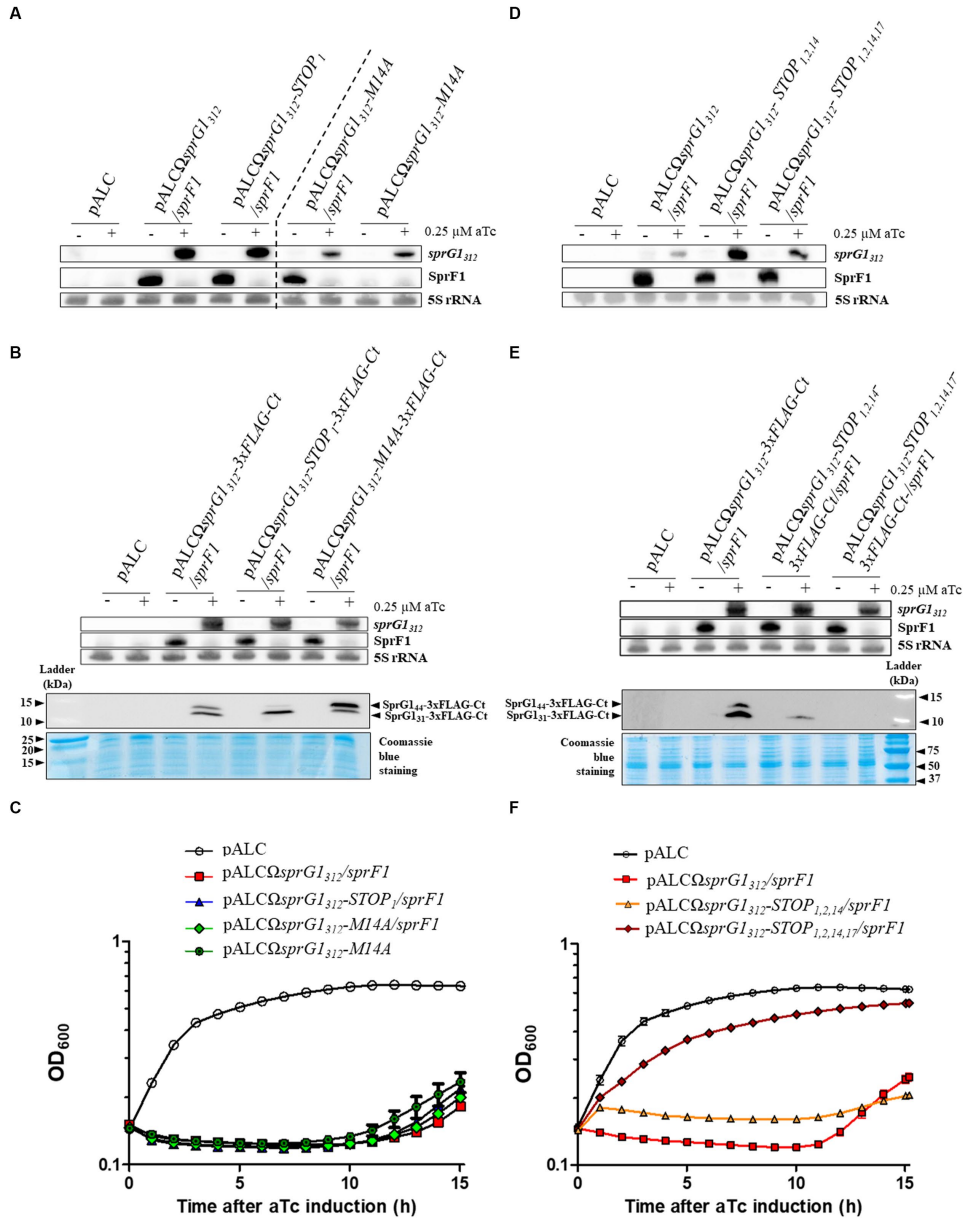
*SprG1_312_* overexpression triggers *Staphylococcus aureus* growth inhibition caused by either of the encoded peptide. **(A–C)** *S. aureus* N315Δ*sprG1/sprF1* strains carrying pALC, pALCΩ*sprG1_312_/sprF1*, pALCΩ*sprG1_312_-STOP_1_/sprF1*, pALCΩ*sprG1_312_-M14A/sprF1*, pALCΩ*sprG1_312_-M14A*, pALCΩ*sprG1_312_-3xFLAG-Ct/sprF1*, pALCΩ*sprG1_312_-STOP_1_-3xFLAG-Ct /sprF1*, pALCΩ*sprG1_312_-M14A-3xFLAG-Ct/sprF1* were cultivated in MH medium until the exponential growth phase and incubated in the absence (−) or presence (+) of 0.25 μM aTc. **(A,B)** After RNA extraction, northern blot analysis was done on *sprG1_312_*, *sprG1_312_* mutants and SprF1 expression with 5S rRNA used as the loading control. **(B)** After protein extraction, the expression of the *sprG1_312_*-encoded flagged peptides, SprG1_44_-3xFLAG-Ct and SprG1_31_-3xFLAG-Ct, was analyzed by western blots by using anti-FLAG antibodies. Coomassie blue staining was used as the loading control. **(C)** Growth kinetics of *S. aureus* strains after aTc induction. Error bars show the means and standard deviations of three biological replicates. **(D–F)** *S. aureus* N315Δ*sprG1/sprF1* strains carrying pALC, pALCΩ*sprG1_312_/sprF1*, pALCΩ*sprG1_312_-STOP_1,2,14_/sprF1*, pALCΩ*sprG1_312_-STOP_1,2,14,17_/sprF1*, pALCΩ*sprG1_312_-3xFLAG-Ct/sprF1*, pALCΩ*sprG1_312_-STOP_1,2,14_-3xFLAG-Ct /sprF1*, pALCΩ*sprG1_312_-STOP_1,2,14,17_-3xFLAG-Ct/sprF1* were cultivated in MH medium until the exponential growth phase and incubated in the absence (−) or presence (+) of 0.25 μM aTc. **(D,E)** After RNA extraction, northern blot analysis was done on *sprG1_312_*, *sprG1_312_* mutants and SprF1 expression with 5S rRNA used as the loading control. **(E)** After protein extraction, the expression of the *sprG1_312_*-encoded flagged peptides, SprG1_44_-3xFLAG-Ct and SprG1_31_-3xFLAG-Ct, was analyzed by western blots by using anti-FLAG antibodies. Coomassie blue staining was used as the loading control. **(F)** Growth kinetics of *S. aureus* strains after aTc induction. Error bars show the means and standard deviations of three biological replicates (*n* = 3).

In a previous study, we demonstrated that *sprG1* mRNA is expressed as two transcripts differing at their 5′-ends, the *sprG1_312_* major form and the *sprG1_439_* minor form, both encoding SprG1_31_ and SprG1_44_, in the *S. aureus* N315 strain (Supplementary Figure S1). Moreover, we showed that the overexpression of *sprG1_439_*-encoded peptides inhibits *S. aureus* growth and induces cell death (Pinel-Marie et al., 2014). In this work, focusing on the predominant *sprG1_312_* mRNA, we used a model of *sprG1_312_*-encoded peptides overexpression in the *S. aureus* N315 strain, which was deleted for the endogenous *sprG1/sprF1* locus. First, we investigated whether the overexpression of *sprG1_312_*-encoded peptides also hampers *S. aureus* growth. To achieve this, we cloned *sprG1_312_* into pALC, allowing the anhydrotetracycline (aTc)-inducible expression of SprG1_44_ and SprG1_31_, with (pALCΩ*sprG1_312_/sprF1*) or without (pALCΩ*sprG1_312_*) the SprF1 antitoxin, expressed under its own promoter (Table 1; Supplementary Figure S1). In the absence of SprF1 antitoxin, we failed to obtain clones in *S. aureus*, likely due to transcriptional leakage of the aTc-inducible promoter and the high toxicity of SprG1_44_ and SprG1_31_ (Table 1; Supplementary Figure S1). To evaluate the toxicity of SprG1_31_ and SprG1_44_ individually, we constructed the pALCΩ*sprG1_312_-STOP_1_* mutant, in which the AUG54 initiation codon was replaced with a UAA termination codon, and pALCΩ*sprG1_312_-M14A* mutant, in which the AUG93 initiation codon was replaced with a GCC alanine codon, expressing only SprG1_31_ or SprG1_44_, respectively (Supplementary Figures S1, S2; Table 1). Similar to pALCΩ*sprG1_312_*, no clones were obtained in *S. aureus* for the pALCΩ*sprG1_312_-STOP_1_* mutant as oppose to the pALCΩ*sprG1_312_-M14A* mutant in the absence of SprF1 (Table 1; Supplementary Figure S1). This suggests that SprG1_31_ is more toxic to *S. aureus* compared to SprG1_44_, which aligns with the lower MIC of SprG1_31_ on *S. aureus* than the MIC of SprG1_44_ (Pinel-Marie et al., 2014). We performed northern blots to validate the production of each overexpressed *sprG1_312_* mRNA after aTc induction and to assess SprF1 RNA (Figure 1A). We observed a significant decrease in SprF1 RNA level strongly dropped after aTc induction, suggesting a SprF1 RNA degradation mediated by RNases. To confirm the expression of SprG1_31_ and SprG1_44_ in all our constructs, we next added a 3xFLAG sequence in-frame ahead of the predicted termination codon (Table 2). Northern blots confirmed the expression of *sprG1_312_*-*3xFLAG-Ct* RNA and the reduction of SprF1 RNA level for each construct after aTc induction (Figure 1B). As expected, western blots revealed that the pALCΩ*sprG1_312_-3xFLAG-Ct/sprF1* construct expressed both peptides, with a higher expression of SprG1_31_, the pALCΩ*sprG1_312_-STOP_1_-3xFLAG-Ct/sprF1* mutant expressed mainly SprG1_31_ while the pALCΩ*sprG1_312_-M14A-3xFLAG-Ct/sprF1* mutant predominantly expressed SprG1_44_ (Figure 1B). Without aTc, *S. aureus* cells containing each of the constructs grown similarly to the cells containing the empty vector (Supplementary Figures S3A, S3B). In the presence of aTc, the induced transcription of *sprG1_312_*, *sprG1_312_*-*3xFLAG-Ct*, *sprG1_312_*-*STOP_1_*, *sprG1_312_- STOP_1_-3xFLAG-Ct*, *sprG1_312_*-*M14A* RNAs inhibited *S. aureus* growth contrary to the *sprG1_312_*-*M14A-3xFLAG-Ct* RNA (Figure 1C; Supplementary Figure S3C). However, 10 h after aTc induction, the *S. aureus* growth resumed, indicating that the overexpression of toxins did not lead to the death of the entire bacterial population. Moreover, this result seems to confirm the higher toxicity of SprG1_31_ compared to SprG1_44_ towards *S. aureus*.

To demonstrate that *sprG1_312_* inhibits *S. aureus* growth due to its encoding of toxic peptides, we generated pALCΩ*sprG1_312_-STOP_1,2,14_/sprF1* and pALCΩ*sprG1_312_- STOP_1,2,14_*-*3xFLAG-Ct/sprF1* constructs in which the AUG54, GUG57 and AUG93 initiation codons were replaced with a UAA termination codon (Supplementary Figures S1, S2). Through northern blot analysis, we confirmed the expression of each overexpressed *sprG1_312_* RNA and observed a decrease in SprF1 RNA level after aTc induction (Figures 1D,E). Surprisingly, during western blot analysis, we detected the presence of a flagged peptide smaller than SprG1_31_, which remained toxic to *S. aureus* after aTc induction (Figures 1E,F). We deduced that the amino acids sequence for this additional peptide is MLQFGLFLIALIGLVIKLIELSNKK. Consequently, we decided to construct the pALCΩ*sprG1_312_- STOP_1,2,14,17_*-*3xFLAG-Ct/sprF1* mutant by replacing the ATT102 isoleucine codon by a UAA termination codon to prevent the production of the additional flagged peptide by ribosomes likely starting from the AUG111 initiation codon (Supplementary Figures S1, S2). For this construct, we confirmed, after aTc induction: i) the expression of *sprG1_312_* RNA and the reduction of SprF1 RNA level through northern blot, ii) the absence of flagged peptide expression through western blot and, iii) the restoration of *S. aureus* growth (Figures 1D–F). Moreover, *SprG1_312_* expression also slightly impaired growth independently of the translation products (Figure 1F), suggesting toxicity associated with the *sprG1_312_* RNA itself. Additionally, we verified that *S. aureus* cells carrying each mutant grew similarly to the cells carrying the empty vector in the absence of aTc (Supplementary Figure S3D).

Altogether, our results provide strong evidence that *sprG1_312_* overexpression inhibits *S. aureus* growth due to the presence of two toxic peptides, SprG1_31_ and SprG1_44_. Moreover, the production of either of these peptides leads to growth arrest, which is alleviated in the presence of the SprF1 antitoxin. Notably, our findings indicate that SprG1_31_ exhibits higher toxicity towards *S. aureus* compared to SprG1_44_. Additionally, we have identified that the MLQFGLFLIAL IGLVIKLIELSNKK peptide alone is sufficient to induce growth arrest in *S. aureus*.

### Sequence requirements for SprG1_31_ toxicity

**Figure 2:**
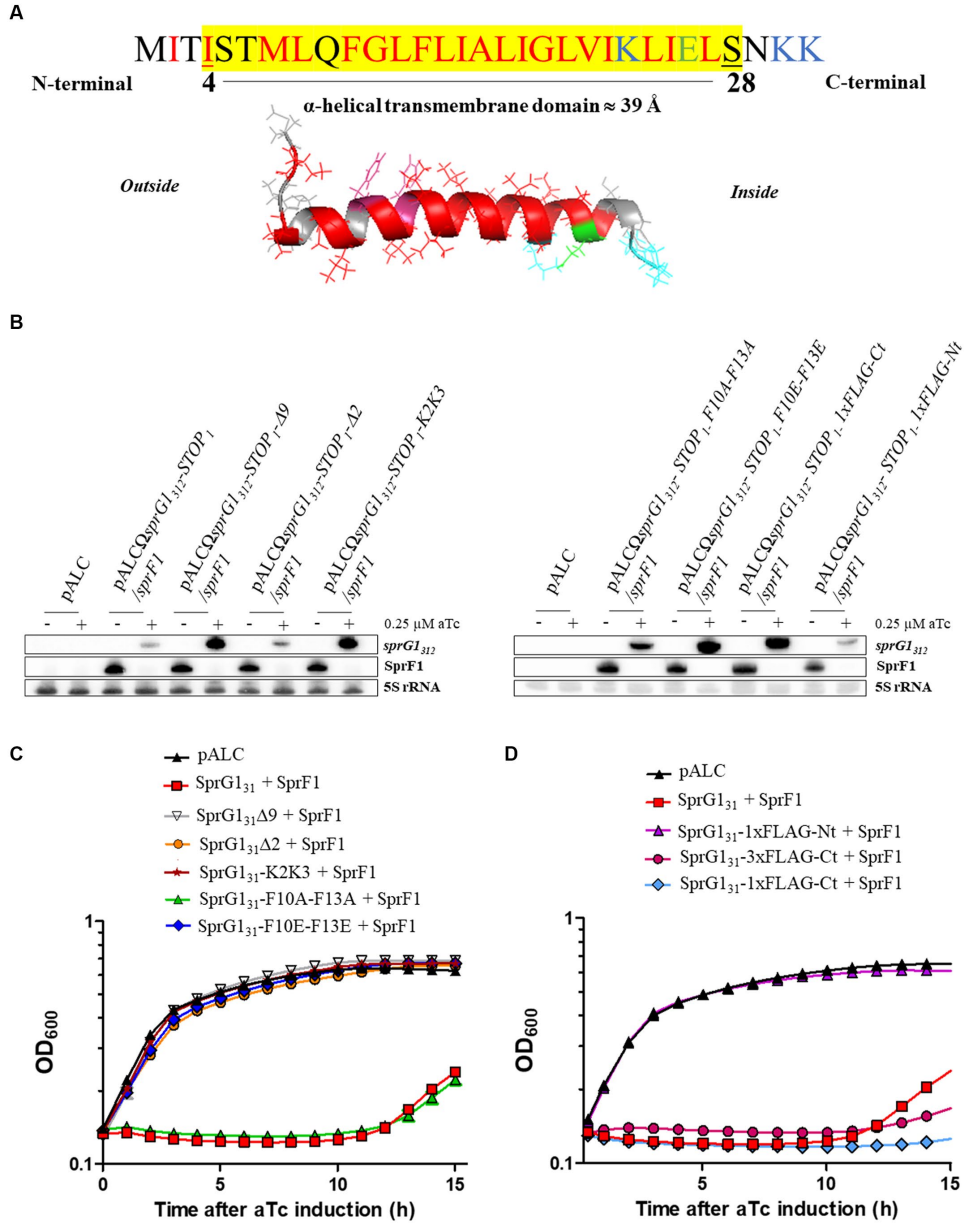
Toxicity of SprG1_31_ mutants in *Staphylococcus aureus*. **(A)** Schematic representations of the SprG1_31_ toxin with the α-helical domain ranging from I4 to S28. The putative transmembrane domain, highlighted in yellow, is predicted using the DeepTMHMM algorithm along with the orientation of the computed *in silico* model of α-helix from the inside to the outside of the bacterial membrane (Hallgren et al., 2022). Hydrophobic amino acids are shown in red, negatively charged amino acids in green and positively charged amino acids in blue. **(B,C)** *S. aureus* N315Δ*sprG1/sprF1* strains carrying pALC, pALCΩ*sprG1_312_-STOP_1_/sprF1* (SprG1_31_ + SprF1), pALCΩ*sprG1_312_-STOP_1_-Δ9/sprF1* (SprG1_31_Δ9 + SprF1), pALCΩ*sprG1_312_-STOP_1_-Δ2/sprF1* (SprG1_31_Δ2 + SprF1), pALCΩ*sprG1_312_-STOP_1_-K2K3/sprF1* (SprG1_31_-K2K3 + SprF1), pALCΩ*sprG1_312_-STOP_1_-F10A-F13A/sprF1* (SprG1_31_-F10A-F13A + SprF1), pALCΩ*sprG1_312_-STOP_1_-F10E-F13E/sprF1* (SprG1_31_-F10E-F13E + SprF1), pALCΩ*sprG1_312_-STOP_1_ 1xFLAG-Nt/sprF1* (SprG1_31_-1xFLAG-Nt + SprF1), pALCΩ*sprG1_312_-STOP_1_ 1xFLAG-Ct/sprF1* (SprG1_31_-1xFLAG-Ct + SprF1) and pALCΩ*sprG1_312_-3xFLAG-Ct-STOP_1_/sprF1* (SprG1_31_-3xFLAG-Ct + SprF1) were cultivated in MH medium until the exponential growth phase and incubated in the absence (−) or presence (+) of 0.25 μM aTc. **(B)** After RNA extraction, northern blot analysis was done on *sprG1_312_*, *sprG1_312_* mutants and SprF1 expression with 5S rRNA used as the loading control. **(C,D)** Growth kinetics of *S. aureus* strains after aTc induction. Error bars show the means and standard deviations of three biological replicates (*n* = 3).

We have previously solved the 3D structure of SprG1_31_ using NMR (Nonin-Lecomte et al., 2021). We demonstrated that SprG1_31_ possesses an α-helical domain about 39 Å long, ranging from I4 to S28 (Figure 2A). Additionally, we observed that the three N-terminal MIT residues and the three hydrophilic C-terminal NKK residues are unstructured (Figure 2A). To investigate the specific amino acids responsible for SprG1_31_ toxicity, we engineered five mutants expressing truncated peptides. These mutants included SprG1_31_Δ9 and SprG1_31_Δ2, where the last nine and the two residues were deleted, respectively. Furthermore, we placed the C-terminal two lysine residues at the N-terminal domain in the SprG1_31_-K2K3 mutant. Additionally, we replaced the two phenylalanine residues at positions 10 and 13 with either alanine residues (SprG1_31_-F10A-F13A) or glutamic acid residues (SprG1_31_-F10E-F13E). We successfully generated these mutants in the presence or absence of the SprF1 antitoxin, demonstrating that they exhibited reduced toxicity compared to wild-type SprG1_31_. Through northern blot analysis, we confirmed the expression of each overexpressed RNA upon aTc induction, as well as the decline in SprF1 RNA levels in the mutants expressing the antitoxin (Figure 2B; Supplementary Figure S4A). In the absence of aTc, all mutant strains exhibited growth rates similar to the strain carrying the empty vector (Supplementary Figure S4B). To assess the impact of C-terminal charged part of SprG1_31_, we deleted the two last lysines (SprG1_31_∆2 mutant) and the nine last amino acids (SprG1_31_∆9 mutant). Both mutants produced peptides with a net global charge close to zero (Table 2). The overexpression of these two truncated peptides did not exert toxicity effect on *S. aureus* growth after aTc induction in presence of SprF1 (Figure 2C). When we removed *sprF1* gene from the constructs, the level of SprG1 peptides must be higher because SprF1 RNA prevents the translation of s*prG1_312_* RNA (Pinel-Marie et al., 2014). We showed that removing *sprF1* gene had no impact on the lack of toxicity to the SprG1_31_∆9 mutant; even a higher level of SprG1_31_ peptide deleted from its nine last residues remains non-toxic. However, a higher level of SprG1_31_ deleted from its two last lysines is toxic for the bacteria (Supplementary Figure S5). We can conclude that the two last lysines are involved in the SprG1_31_ toxicity but are not necessary. On the contrary, the nine last charged residues are necessary for SprG1_31_ toxicity. Several studies showed that the global charge of antimicrobial peptides is important, but above all the position of the positive charges (Zhang et al., 2016). In order to assess if the position of the two last cationic lysines of SprG1_31_ is crucial for the peptide toxicity, we transferred them in the N-terminal part. Interestingly, toxicity was again abolished upon aTc induction, regardless of the presence or absence of SprF1 (Figure 2C; Supplementary Figure S5). Keeping the cationic charges in the C-terminal part is therefore a key property for peptide toxicity. Finally, as phenylalanine residues are known to be crucial for peptide binding to the bacterial membrane (Victor et al., 1999; Van Kan et al., 2003; Shahmiri et al., 2017), we examined the significance of the two phenylalanine residues (F10 and F13) in SprG1_31_ toxicity. Surprisingly, replacing both phenylalanine residues with alanine residues (SprG1_31_-F10A-F13A) did not affect SprG1_31_ toxicity on *S. aureus* growth upon aTc induction (Figure 2C). However, disturbing the hydrophobicity and likely the α-helical transmembrane domain by replacing both phenylalanine residues by glutamic acid residues [SprG1_31_-F10E-F13E, resulting in a net charge close to zero (Table 2)], led to the abolishment of SprG1_31_ toxicity upon aTc induction, irrespective of the absence or presence of SprF1 (Figure 2C; Supplementary Figure S5).

We also examined the impact of adding a 1xFLAG sequence at either the N-terminal domain (SprG1_31_-1xFLAG-Nt) or at the C-terminal domain (SprG1_31_-1xFLAG-Ct) compared to the SprG1_31_-3xFLAG-Ct construct (Table 2). As shown in the Figure 2B, the flagged *sprG1_312_* RNAs were overexpressed, and the SprF1 RNA level decreased after aTc induction. Note that none of these mutants exhibited a growth phenotype without aTc (Supplementary Figure S6A). However, in the presence of aTc, only the presence of the 1xFLAG sequence at the N-terminal domain resulted in the removal of SprG1_31_ toxicity (Figure 2D). To investigate whether the lack of toxicity in SprG1_31_-1xFLAG-Nt was due to a defect in the peptide’s insertion into the *S. aureus* membrane, we performed cell fractionation experiments. The results showed that the SprG1_31_-1xFLAG-Nt peptide was present in the membrane, similar to the SprG1_31_-3xFLAG-Ct peptide (Supplementary Figure S6B). This suggests that the presence of charged amino acids at the N-terminal domain does not affect the insertion of SprG1_31_ into the membrane, but rather impairs its toxicity. To gain deeper understanding of the lack of toxicity when the 1xFLAG sequence is positioned at the N-terminal domain of SprG1_31_, we used the DeepTMHMM algorithm^1^ to predict the orientation of SprG1_31_ in the bacterial membrane (Hallgren et al., 2022). The results showed that adding the 1xFLAG sequence at the C-terminal domain or at the N-terminal domain does not impact the orientation of SprG1_31_ in the membrane, with the N-terminal domain predicted to be localized in the extracellular medium (Figure 2A). This indicates that the loss of toxicity observed for the SprG1_31_-1xFLAG-Nt peptide is not due to a modification of the orientation of SprG1_31_. However, the results also showed that removing or displacing the two last lysine residues in the SprG1_31_Δ2 and SprG1_31_-K2K3 mutants modifies the orientation of SprG1_31_. Consequently, keeping the C-terminal part in the cytosol may promote SprG1_31_ toxicity as SprG1_31_Δ2 mutant is toxic only in the presence of SprF1 but is probably not a key feature for the peptide toxicity.

Overall, our findings provide strong evidence that the toxicity of SprG1_31_ is a result of the combination of its α-helical transmembrane domain and charged amino acids positioned at the C-terminal domain.

### SprG1_44_ and SprG1_31_ induce membrane depolarization followed by intracellular ATP depletion responsible for *S. aureus* growth inhibition

**Figure 3:**
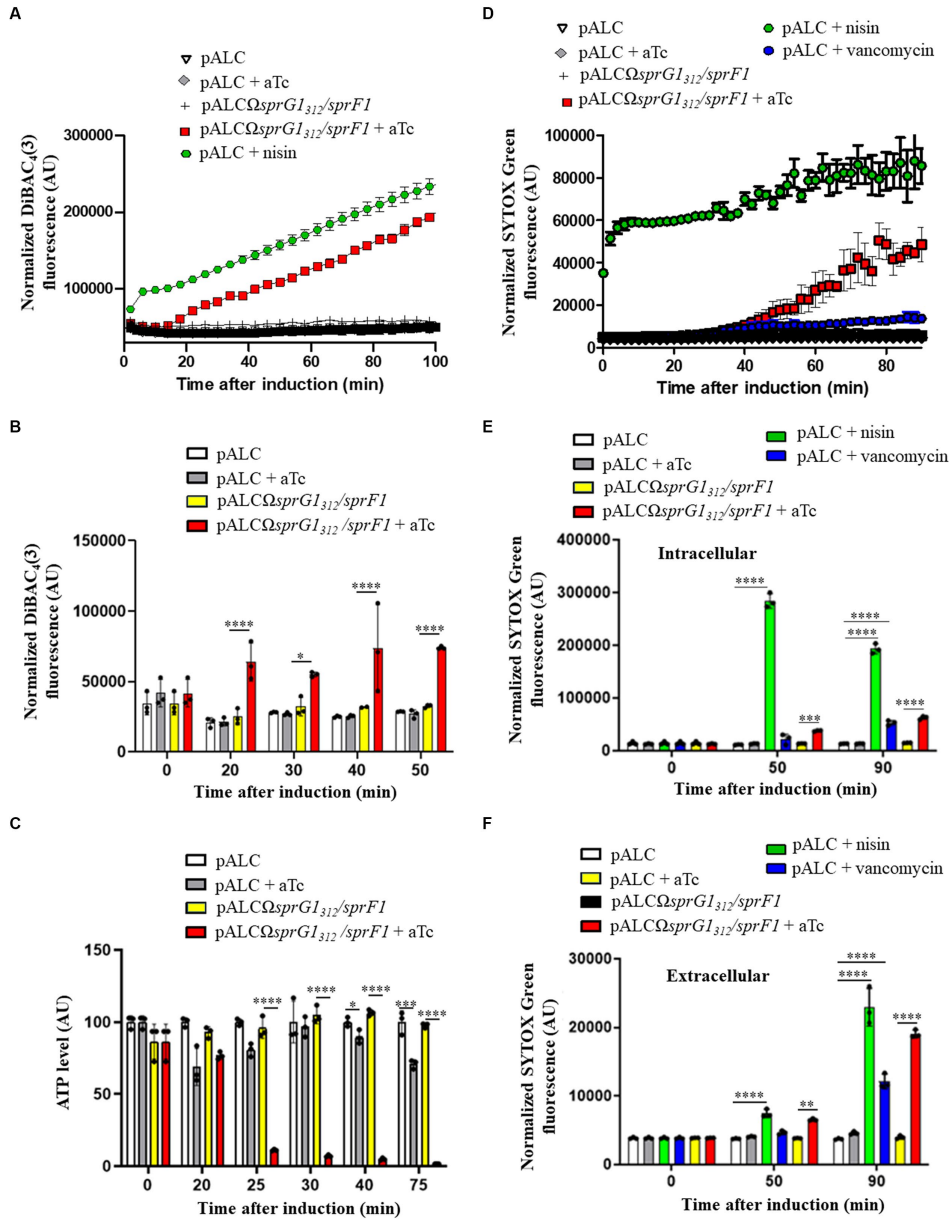
*SprG1_312_* overexpression triggers membrane depolarization and a drop in intracellular ATP leading to membrane permeabilization in *Staphylococcus aureus*. **(A)** *S. aureus* N315Δ*sprG1/sprF1* strains carrying pALC and pALCΩ*sprG1_312_/sprF1* were cultivated in MH medium until the exponential growth phase, resuspended in PBS supplemented with 25 mM glucose, stained with 0.5 μg/mL DiBAC_4_(3) for 10 min at 37°C to stabilize fluorescence and incubated in absence or presence of 0.25 μM aTc or 12.5 μg/mL nisin used as the positive control. DiBAC_4_(3) fluorescence was measured in a microplate reader and normalized with OD_600_. **(B)** *S. aureus* N315Δ*sprG1/sprF1* strains carrying pALC and pALCΩ*sprG1_312_/sprF1* were cultivated in MH medium until the exponential growth phase and incubated in absence or presence of 0.25 μM aTc or 12.5 μg/mL nisin used as the positive control. At each time point, bacteria were resuspended in PBS and stained with 0.5 μg/mL DiBAC_4_(3). DiBAC_4_(3) fluorescence values were normalized with OD_600_. **(C)** *S. aureus* N315Δ*sprG1/sprF1* strains carrying pALC and pALCΩ*sprG1_312_/sprF1* were cultivated in MH medium until the exponential growth phase and incubated in absence or presence of 0.25 μM aTc. At each time point, bacteria were resuspended in MH and incubated with BacTiter-Glo Microbial Cell Viability Assay. Luminescence values were normalized with OD_600_. **(D)** *S. aureus* N315Δ*sprG1/sprF1* strains carrying pALC and pALCΩ*sprG1_312_/sprF1* were cultivated in MH medium until the exponential growth phase, resuspended in PBS supplemented with 25 mM glucose, stained with 5 μM SYTOX Green for 25 min at 37°C to stabilize fluorescence and incubated in absence or presence of 0.25 μM aTc, 12.5 μg/mL nisin or 28 μg/mL vancomycin, used as the positive controls. SYTOX Green fluorescence values were normalized with OD_600_. **(E,F)** *S. aureus* N315Δ*sprG1/sprF1* strains carrying pALC and pALCΩ*sprG1_312_/sprF1* were cultivated in MH medium until the exponential growth phase and incubated in absence or presence of 0.25 μM aTc, 12.5 μg/mL nisin or 28 μg/mL vancomycin, used as the positive controls. At each time point, supernatants (extracellular fraction) or bacteria resuspended in PBS (intracellular fraction) were stained with 2.5 μM SYTOX Green. SYTOX Green fluorescence values were normalized with OD_600_. Error bars show the means and standard deviations of three biological replicates (*n* = 3). Statistical significance was calculated with the two-way ANOVA with Tukey’s correction. *p < 0.05; **p < 0.01; ***p < 0.001; ****p < 0.0001. AU refers to arbitrary unit.

In our previous study (Pinel-Marie et al., 2014), we demonstrated that ectopic overexpression of the two *sprG1_439_*-encoded peptides leads to cell death in *S. aureus* by disturbing membrane integrity within 1 h of induction. In this work, we aimed to establish the sequence of toxic events induced by SprG1_44_ and SprG1_31_ overexpression on the bacterial membrane. We first examined the effect of these peptides on membrane depolarization using the potential-sensitive probe, bis-(1,3-dibutylbarbituric acid)-trimethine oxonol [DiBAC_4_(3)], which crosses depolarized membranes and emits fluorescence when it binds to intracellular hydrophobic sites (Wu et al., 2017). To determine the timing of these toxic events, we followed membrane depolarization after aTc induction using a microplate assay in PBS supplemented with 25 mM glucose (Figure 3A) (Clementi et al., 2014). The positive control, the pore-forming peptide Nisin, immediately increased DiBAC_4_(3) fluorescence within 2 min after incubation, while SprG1_44_ and SprG1_31_ overexpression caused an increase in DiBAC_4_(3) fluorescence within 20 min upon aTc induction (Figure 3A). To confirm this kinetic, we performed time-point measurements in MH medium. The times shown in Figures 3B,C refer to the time between aTc induction and measurement of fluorescence/luminescence and OD_600_ in the microplate reader. We confirmed here that the membrane depolarization was statistically significant 20 min after the overexpression of SprG1_44_ and SprG1_31_ (Figure 3B; Supplementary Figure S7A). Interestingly, this perturbation of the membrane was accompanied by a decrease in OD_600nm_ values in MH medium or PBS supplemented with 25 mM glucose, observed 15–20 min after aTc induction (Supplementary Figure S8). We then investigated whether these events were followed by a reduction in intracellular ATP level, as seen in other type I toxins, such as HokB, ZorO and DinQ type I toxins (Weel-Sneve et al., 2013; Wilmaerts et al., 2018; Bogati et al., 2022b). As expected, nisin induced a significant drop in intracellular ATP levels, followed by an increase in extracellular ATP levels after 20 min incubation, due to its ability to form large pores in the *S. aureus* membrane (between 2.0 to 2.5 nm in model membranes) (Supplementary Figures S7B) (Wiedemann et al., 2004). Similarly, SprG1_44_ and SprG1_31_ caused a significant reduction in intracellular ATP levels after 25 min of overexpression (Figure 3C). However, there was no significant difference in extracellular ATP levels after SprG1_44_ and SprG1_31_ induction (Supplementary Figure S7C).

Based on these results, we can conclude that overexpression of SprG1_44_ and SprG1_31_ inhibits *S. aureus* growth though membrane depolarization and consequent depletion of intracellular ATP.

### SprG1_44_ and SprG1_31_ permeabilize the *S. aureus* membrane without large pore formation leading to cell leakage

In addition to examining membrane depolarization, we also assessed the effect of SprG1_44_ and SprG1_31_ overexpression on *S. aureus* membrane permeabilization using the SYTOX green dye. This dye enters cells through pores with a radius larger than 0.5 to 0.7 nm, binds to DNA, and fluoresces, allowing us to monitor membrane permeabilization (Bodénès et al., 2019). We conducted the same kinetics in a microplate in PBS supplemented with 25 mM glucose for comparison with the membrane depolarization assays. As expected, the positive control, nisin, induced a strong and rapid *S. aureus* membrane permeabilization, leading to a significant increase in SYTOX green fluorescence within 2 min after incubation (Figure 3D), consistent with previous studies (Jensen et al., 2020). In contrast, SprG1_44_ and SprG1_31_ caused a gradual increase of SYTOX green fluorescence starting from 40 min after aTc induction (Figure 3D), indicating a progressive disruption of the *S. aureus* membrane, independent of the formation of large pores. Moreover, vancomycin, which inhibits cell wall synthesis but does not directly form pores in the *S. aureus* membrane (Watanakunakorn, 1984), resulted in a weaker increase in SYTOX green fluorescence compared to nisin and *sprG1_312_*-encoded peptides (Figure 3D). To determine whether the membrane permeabilization induced by SprG1_44_ and SprG1_31_ leads to cell lysis, we measured the SYTOX green fluorescence in both intracellular and extracellular fractions. As shown before, nisin directly permeabilized the bacterial membrane to reach a stable and high fluorescence of SYTOX green (Figure 3D). We next measured the SYTOX green fluorescence in the intracellular fraction 50 min after nisin addition as this is when an increase of SYTOX green fluorescence induced by the overexpression of SprG1_31_ and SprG1_44_ is expected (Figure 3D). We confirmed here that the bacterial cells are still permeabilized 50 min after nisin addition, as evidenced by a significant and maximal increase in SYTOX green fluorescence in the intracellular fraction (Figure 3E). However, the maximal increase in SYTOX green fluorescence for the extracellular fraction was observed 90 min after nisin incubation (Figure 3F), indicating a delay between membrane permeabilization and cell leakage. Nisin has been shown to form pores in the bacterial cell membrane as a direct effect after incubation and also induces autolysis (Bierbaum and Sahl, 1985; Severina et al., 1998). Similarly, vancomycin, which induces autolysis without forming pores (Hsu et al., 2011), resulted in a significant increase in SYTOX green fluorescence in both the intracellular and extracellular fractions 90 min after incubation, though lower than that of nisin (Figures 3E,F). This suggests that vancomycin-induced autolysis leads to membrane permeabilization and, consequently, cell leakage. Likewise, SprG1_44_ and SprG1_31_ triggered a membrane permeabilization accompanied by cell leakage 50 and 90 min after induction. The significant increase in SYTOX green fluorescence in both the intracellular and extracellular fractions occurred simultaneously and maximally 90 min after induction (Figures 3E,F).

Taken together, our results demonstrate that SprG1_44_ and SprG1_31_, when overexpressed, induce inhibition of *S. aureus* growth by first causing membrane depolarization and depletion of intracellular ATP. Subsequently, they induce membrane permeabilization, leading to cell leakage. Unlike nisin, our findings strongly suggest that SprG1_44_ and SprG1_31_ are not capable of forming large pores in the *S. aureus* membrane.

### SprG1_44_ and SprG1_31_ display mesosome-like structures on the *S. aureus* membrane and promote cell lysis

**Figure 4:**
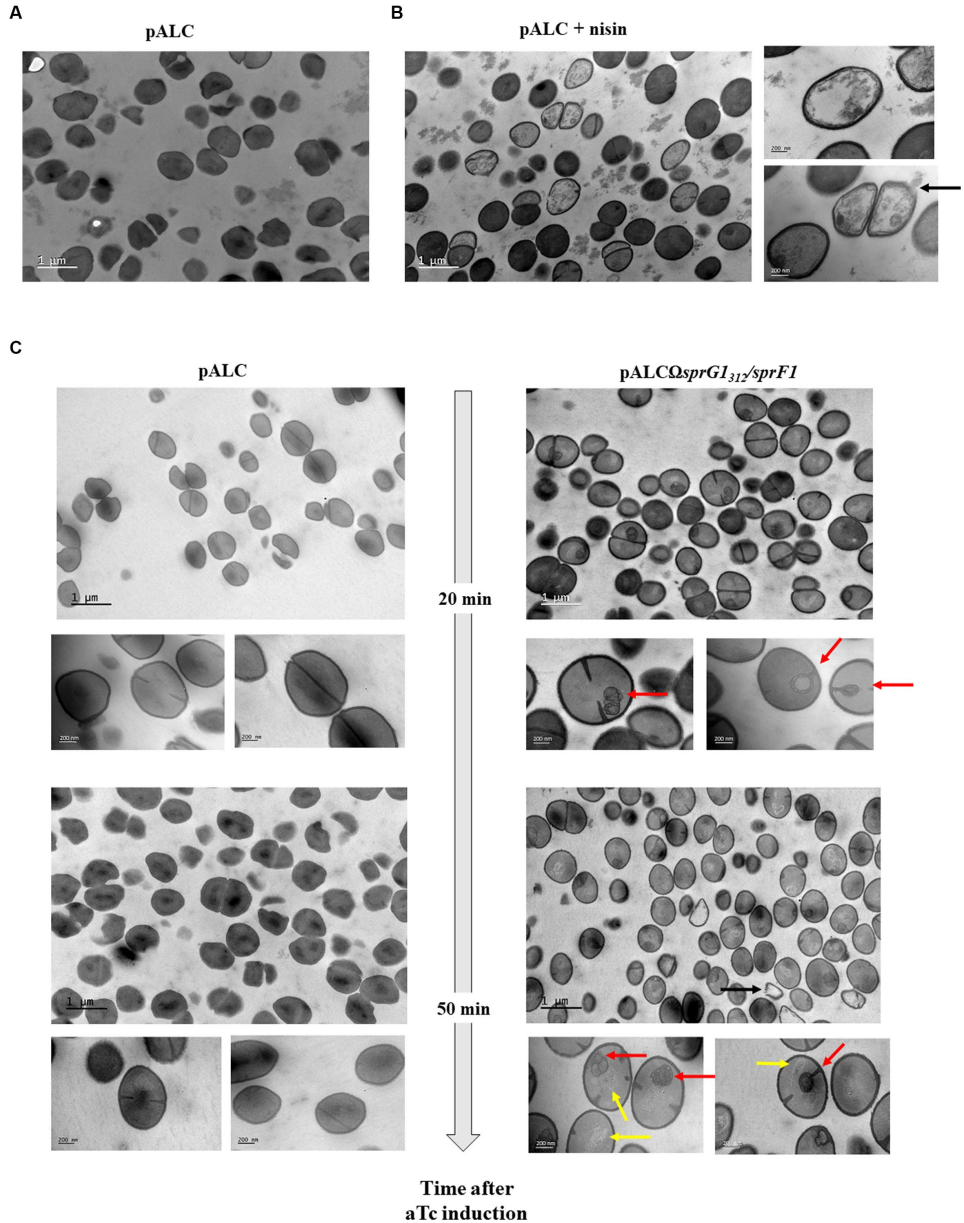
*SprG1_312_* overexpression causes mesosomes-like structures. *S. aureus* N315Δ*sprG1/sprF1* strains carrying pALC and pALCΩ*sprG1_312_/sprF1* were cultivated in MH medium until the exponential growth phase and incubated in absence **(A)** or presence of 12.5 μg/mL nisin for 30 min used as the positive control **(B)** or 0.25 μM aTc for 20 or 50 min **(C)**. *S. aureus* morphology analysis was investigated with transmission electron microscopy. Bars represent 200 nm or 1 μm. Black arrow shows empty bacteria with partly disintegrated cell wall. Red arrows show intracellular mesosomal structures located either in the bacterial septum either at the bacterial pole. Yellow arrows show changes in the shape and structure of nucleoids which appears as slight filaments in the bacterial cytosol.

As previously described, when SprG1_44_ and SprG1_31_ are overexpressed in *S. aureus*, they induce membrane perturbations leading to growth inhibition. To further investigate the effects of these peptides on bacterial morphology, we used transmission electron microscopy (TEM) to visualize bacterial cells at 20 min and 50 min after aTc induction, when the membrane is depolarized and permeabilized. In the negative control (pALC), bacterial cells showed intact cells walls and membranes, and the nucleoid appeared as a condensed dark gray structure within the bacteria (Figure 4A). As expected, the positive control nisin caused lysis of *S. aureus*, resulting in empty bacteria with destroyed membranes and, in some cases, holes in the cell wall (Figure 4B). In contrast to nisin, TEM images of cells 20 min after SprG1_44_ and SprG1_31_ induction revealed the presence of intracellular mesosome-like structures in significant number of bacteria (Figure 4C). These mesosome-like structures were frequently located in the bacterial septum, suggesting a blockage of *S. aureus* division consistent with the growth inhibition induced by SprG1_44_ and SprG1_31_ (Figure 4C). Likewise, 50 min after induction, we distinguished mesosome-like structures, but also changes in nucleoid shape and structure appearing as light filaments located to bacterial cytosol (Figure 4C). Interestingly, no significant differences in cell wall thickness were observed when toxins were overexpressed (Supplementary Figure S9), indicating that the observed morphological changes were primarily related to membrane perturbations rather than alterations in cell wall structure.

Based on these observations, we conclude that SprG1_44_ and SprG1_31_ overexpression promotes the formation of mesosome-like structures, mainly located in the bacterial septum, and induce changes in nucleoid shape and structure. These morphological changes are consistent with the membrane perturbations induced by SprG1_44_ and SprG1_31_, and subsequent lysis of *S. aureus*.

## Discussion

In this study, we focused on understanding the sequence requirements and mechanisms of action of two membrane-associated type I toxins, SprG1_44_ and SprG1_31_, which are encoded by the *sprG1/sprF1* locus in *S. aureus*. Although we were unable to identify any specific phenotype when the *sprG1* gene was deleted, we devised a strategy to overproduce SprG1_44_ and SprG1_31_ peptides using the pALC plasmid with an aTc-inducible promoter, derived from the predominant *sprG1_312_* mRNA in *S. aureus*. Through our experiments, we observed that SprG1_31_ exhibited higher toxicity towards *S. aureus* compared to SprG1_44_. This was evident from the fact that only overproduction of SprG1_44_ in the absence of SprF1 antitoxin allowed bacterial viability, while the overproduction of SprG1_31_ without SprF1 led to *S. aureus* death. This outcome aligns with the previously established antimicrobial activity of these two peptides (Pinel-Marie et al., 2014).

Despite real diversity in terms of length and amino acids sequence, the membrane-associated type I toxins share common features such as their relatively short length (less than 60 amino acids), the presence of a putative α-helical transmembrane domain, and the occurrence of positively charged residues primarily located in the C-terminal part (Nonin-Lecomte et al., 2021). These positively charged residues likely contribute to the affinity of the toxins for negatively charged bacterial membranes. While these charges are not crucial for the toxicity of Fst (Weaver et al., 2009), they are essential for AapA1 (Korkut et al., 2020). To understand the specific amino acids required for SprG1_31_ toxicity towards *S. aureus*, we conducted a mutagenesis approach. We showed that the α-helical transmembrane domain (residues 4 to 28) and the charged amino acids in the C-terminal part (residues 23 to 31) are crucial for maintaining SprG1_31_ toxicity, even though the membrane localization remained intact. This suggests that the N-terminal domain, predicted to be located in the extracellular medium, is not essential for anchoring SprG1_31_ to lipid bilayers but is necessary for inducing membrane perturbations that lead to toxicity. Notably, the net charge of SprG1_31_ had a minimal impact on its toxicity, as both positive and negative charges had no effect on its toxic properties. Our study further demonstrated the essential role of the α-helical transmembrane toxicity. Substituting the two phenylalanine residues (F10 and F13) in this domain with glutamic acids, located on the same side of the α-helix (Nonin-Lecomte et al., 2021), resulted in the suppression of SprG1_31_ toxicity. This aligns with previous research that showed substitution of specific amino acids in the α-helical transmembrane domain with prolines, which disrupt α-helix conformation, impairs the toxicity of SprG1_44_ (Sur et al., 2022). The α-helical region appears to be critical for membrane interaction in most type I toxins, including Fst, TisB, IsbC, SprA1 and AapA1 (Göbl et al., 2010; Mok et al., 2010; Steinbrecher et al., 2012; Solecki et al., 2015; Korkut et al., 2020). Additionally, specific charged amino acids in the predicted α-helical transmembrane domain are crucial for ZorO-mediated toxicity (Bogati et al., 2022a). We also found that the SprG1_31_ sequence can be shortened to 25 amino acids with a minimum of 16 hydrophobic residues to retain toxicity, likely by maintaining the helix translocation across the membrane.

Here, we aimed to provide a detailed in chronology of the effects induced by the overproduction of SprG1_44_ and SprG1_31_ toxins in *S. aureus*, with the goal of elucidating their primary toxic effects. Despite sharing common features, membrane-associated type I toxins can have distinct mechanisms of action when overexpressed in bacteria. Some toxins primarily induce alterations in the bacterial membrane such as membrane depolarization and/or permeabilization while others induce morphological changes in bacteria before affecting the membrane (Nonin-Lecomte et al., 2021). Membrane depolarization is frequently found after overproduction of type I toxins inducing membrane perturbations. It has been detected by flow cytometry for ShoB (Fozo et al., 2008), IbsC (Fozo et al., 2008) and DinQ (Weel-Sneve et al., 2013) in *E. coli* about 25 min after induction, if we consider the time of incubation before measurement. To detect membrane depolarization, we used a microplate assay, which allowed us to accurately determine the time of membrane depolarization, although it did not provide information on the proportion of depolarized cells. We observed that overproduction of SprG1_44_ and SprG1_31_ toxins led to growth inhibition in *S. aureus* after approximately 20 min of induction, which corresponds approximately to the bacterial division time. Interestingly, this growth inhibition coincided with membrane depolarization, leading to a depletion of intracellular ATP. Notably, we did not detect an increase in extracellular ATP, suggesting that the overproduction of SprG1_44_ and SprG1_31_ may result in the inhibition of ATP synthesis. Furthermore, we hypothesized that these toxins are not capable of forming large pores that would induce ATP leakage, unlike nisin (Wiedemann et al., 2004; Okuda et al., 2013) or HokB type I toxins, which are known to form such pores (Wilmaerts et al., 2019). In our case, we observed that overproduction of SprG1_44_ and SprG1_31_ did not simultaneously cause membrane depolarization and permeabilization, supporting the hypothesis that they do not create large pores in the *S. aureus* membrane. Instead, their mode of action may involve the creation of ionic pores or follow the “carpet” mechanism of membrane disruption. In this mode of action, peptides bind to the membrane surface and induce a detergent-like effect by micellizing the lipid bilayer, similar to antimicrobial peptides (Li et al., 2021) and suggested for the Lpt type I toxin (Maggi et al., 2019). However, the exact mode of action needs to be demonstrated through a comprehensive biophysical approach using chemically-synthetized peptides and membrane models. Additionally, it is essential to identify the exact cellular target(s) of SprG1_44_ and SprG1_31_. Remarkably, our morphological analysis revealed the presence of mesosome-like structures 20 min after SprG1_44_ and SprG1_31_ induction. While these structures were previously considered artifacts, they are now recognized as invaginations of the cytoplasmic membrane often observed after bacterial incubation with antibiotics (Santhana Raj et al., 2007; Morita et al., 2015; Righetto et al., 2023). This study is the first to demonstrate the presence of mesosome-like structures upon type I toxins overproduction. These structures are associated with reactive oxygen species (ROS) formation and may serve as storage locations for hydrogen peroxide (Li et al., 2017). We speculate that the accumulation of SprG1_44_ and SprG1_31_ on the *S. aureus* membrane triggers membrane depolarization, leading to the formation of mesosome-like structures and subsequent ROS production. Recent studies have shown that membrane-depolarizing type I toxins TisB, DinQ, and HokB have the potential to induce ROS formation in *E. coli* (Edelmann and Berghoff, 2019). Furthermore, ROS formation is believed to play a role in cell death induced by antimicrobial peptides that disrupt the membrane (Choi et al., 2015, 2017).

**Figure 5:**
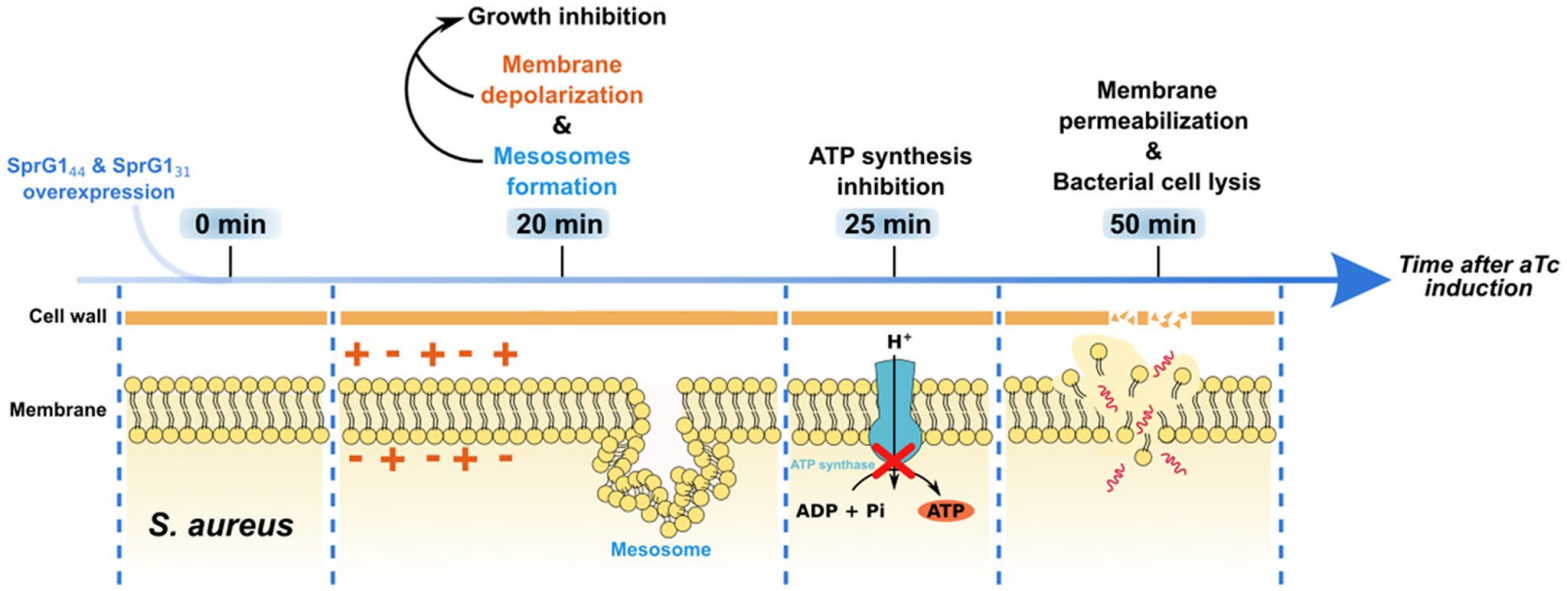
Timeline of toxic effects of SprG1_44_ and SprG1_31_ overexpression on *Staphylococcus aureus*. When overexpressed in *S. aureus*, SprG1_44_ and SprG1_31_ induce membrane depolarization associated with the formation of mesosome-like structures and growth inhibition within 20 min after overexpression. Then, a reduction in intracellular ATP level appears 25 min after peptides overexpression. Together, these events lead to membrane permeabilization and, finally, to lysis of the bacterial cell within 50 min after peptides overexpression.

Overall, we have described two type I toxins that, upon overexpression, primarily induce membrane perturbations in *S. aureus* cells. We have also established a chronology of their toxic effects on bacterial cell (Figure 5). Nevertheless, it is essential to acknowledge that our study, based on ectopic overproduction of SprG1_44_ and SprG1_31_ has limitations, as the toxin concentration is not controlled and is usually higher than what occurs under natural conditions. This could result in off-target effects that may not be observed in natural settings. Moving forward, the next challenge is to identify the conditions under which SprG1_44_ and SprG1_31_ are naturally expressed, and to uncover their potential roles during staphylococcal growth, colonization, and infection. Moreover, the antibacterial potential of peptides derived from type I toxins (Nicolas et al., 2019; Otsuka et al., 2019) demonstrated the proof of concept that toxins can be transformed into potent antibiotics, providing alternatives for eradicating resistant bacteria and persister cells.

## Data availability statement

The original contributions presented in the study are included in the article/Supplementary material, further inquiries can be directed to the corresponding authors.

## Author contributions

LF: Conceptualization, Data curation, Formal analysis, Investigation, Methodology, Software, Writing - original draft, Writing - review & editing. AgB: Data curation, Writing - original draft. EO: Visualization, Writing-review & editing. SD: Data curation. ArB: Conceptualization, Data curation, Formal analysis, Funding acquisition, Investigation, Methodology, Project administration, Resources, Supervision, Validation, Visualization, Writing - review & editing. SC: Conceptualization, Data curation, Formal analysis, Funding acquisition, Investigation, Methodology, Project administration, Resources, Supervision, Validation, Visualization, Writing - review & editing. M-LP-M: Conceptualization, Data curation, Formal analysis, Funding acquisition, Investigation, Project administration, Resources, Supervision, Validation, Visualization, Writing - review & editing, Writing - original draft.

## Footnotes

^1^ https://dtu.biolib.com/DeepTMHMM
