# Supplementary material for: Mechanism of action of sprG1-encoded type I toxins in Staphylococcus aureus: from membrane alterations to mesosome-like structures formation and bacterial lysis
Source: Front Microbiol. 2023 Oct 3;14:1275849. doi: 10.3389/fmicb.2023.1275849 (PMC10579593; doi:10.3389/fmicb.2023.1275849)
Supplement: Supplementary file 1 [file Data_Sheet_1.pdf]

Supplementary Table 1. Strains used in this study.

Strains	Relevant characteristics	References
***E. coli* strain**		
XL1 blue	Δ(ara-leu) 7697 araD139 fhuA ΔlacX74 galK16 galE15 e14-ϕ80dlacZΔM15 recA1 relA1 endA1 nupG rpsL (StrR) rph spoT1 Δ(mrr-hsdRMS-mcrBC)	
***S. aureus* strains**		
RN4220	Restriction-defective derivative of 8325-4	Kreiswirth *et al.*, 1983
N315	Methicillin-resistant *S aureus (*MRSA) strain isolated in 1982 from the pharyngeal smear of a Japanese patient	Okonogi *et al.*, 1989
N315_Δ*sprG1/sprF1*	N315 strain deleted for *sprG1* and *sprF1*	Pinel-Marie *et al.,* 2014

Supplementary Table 2. Plasmids used in this study.

Plasmids	Relevant characteristics	References
pALC2073	Multicopy shuttle vector with the tetracycline-inducible $P_{xyl/tetO}$ promoter and with Cat^R in *S. aureus*	Bateman *et al.*, 2001
pALC2073Ω*sprG1₃₁₂/sprF1*	pALC2073 with *sprG1₃₁₂* under the control of tetracycline-inducible $P_{xyl/tetO}$ promoter and with *sprF1* under the control of its endogenous promoter	This study
pALC2073Ω*sprG1₃₁₂-STOP₁/sprF1*	pALC2073Ω*sprG1₃₁₂/sprF1* with the first AUG codon, encoding to the methionine M_1, mutated to the UAA codon	This study
pALC2073Ω*sprG1₃₁₂-M14A/sprF1*	pALC2073Ω*sprG1₃₁₂/sprF1* with the second AUG codon, encoding to the methionine M_{14}, mutated to the GCC alanine codon	This study
pALC2073Ω*sprG1₃₁₂-M14A*	pALC2073 with *sprG1₃₁₂* with the second AUG codon, encoding to the methionine M_{14}, mutated to the GCC alanine codon	This study
pALC2073Ω*sprG1₃₁₂-STOP₁,₂,₁₄/sprF1*	pALC2073Ω*sprG1₃₁₂/sprF1* with<br>-the first and the second AUG codon, encoding to the methionine M_1 and M_{14}, mutated to the UAA stop codon<br>- the first GUG codon, encoding to the valine V2, mutated to the UAA stop codon	This study
pALC2073Ω*sprG1₃₁₂-STOP₁,₂,₁₄,₁₇/sprF1*	pALC2073Ω*sprG1₃₁₂/sprF1* with<br>-the first and the second AUG codon, encoding to the methionine M_1 and M_{14}, mutated to the UAA stop codon<br>- the first GUG codon, encoding to the valine V2, mutated to the UAA stop codon<br>-the second ATT codon, encoding to the isoleucine I_{17}, mutated to the UAA stop codon	This study
pALC2073Ω*sprG1₃₁₂-3XFlag-Ct/sprF1*	pALC2073Ω*sprG1₃₁₂/sprF1* with a 3XFLAG sequence before the termination codon of the internal coding sequence of $SprG1_{312}$	This study
pALC2073Ω*sprG1₃₁₂-3XFlag-Ct-STOP₁/sprF1*	pALC2073Ω*sprG1₃₁₂-3XFlag-Ct/sprF1* with the first AUG codon, encoding to the methionine M_1, mutated to the UAA codon	This study

pALC2073Ω*sprG1*$_{312}$-*3XFlag-Ct-M14A/sprF1*	pALC2073Ω*sprG1*$_{312}$-*3XFlag-Ct/sprF1* with the second AUG codon, encoding to the methionine M_{14}, mutated to the GCC alanine codon	This study
pALC2073Ω*sprG1*$_{312}$-*3XFlag-Ct-STOP*$_{1,2,14}$*/sprF1*	pALC2073Ω*sprG1*$_{312}$-*3XFlag-Ct/sprF1* with<br>-the first and the second AUG codon, encoding to the methionine M_1 and M_{14}, mutated to the UAA stop codon<br>- the first GUG codon, encoding to the valine V2, mutated to the UAA stop codon	This study
pALC2073Ω*sprG1*$_{312}$-*3XFlag-Ct-STOP*$_{1,2,14,17}$*/sprF1*	pALC2073Ω*sprG1*$_{312}$-*3XFlag-Ct/sprF1* with<br>-the first and the second AUG codon, encoding to the methionine M_1 and M_{14}, mutated to the UAA stop codon<br>- the first GUG codon, encoding to the valine V2, mutated to the UAA stop codon<br>-the second ATT codon, encoding to the isoleucine I_{17}, mutated to the UAA stop codon	This study
pALC2073Ω*sprG1*$_{312}$-*STOP*$_1$*-Δ9/sprF1*	pALC2073Ω*sprG1*$_{312}$-*STOP*$_1$*/sprF1* with the AAG codon, encoding to the lysine K23, mutated to the UAA stop codon	This study
pALC2073Ω*sprG1*$_{312}$-*STOP*$_1$*-Δ2/sprF1*	pALC2073Ω*sprG1*$_{312}$-*STOP*$_1$*/sprF1* with the AAA codon, encoding to the lysine K30, mutated to the UAA stop codon	This study
pALC2073Ω*sprG1*$_{312}$-*STOP*$_1$*-K2K3/sprF1*	pALC2073Ω*sprG1*$_{312}$-*STOP*$_1$*-Δ2/sprF1* with two AAA codons, encoding to two lysine, added after the first AUG codon of SprG1$_{31}$ peptide	This study
pALC2073Ω*sprG1*$_{312}$-*STOP*$_1$*-F10A-F13A/sprF1*	pALC2073Ω*sprG1*$_{312}$-*STOP*$_1$*/sprF1* with the two codons UUC, encoding to the two phenylalanine F10 and F13, mutated to the GCA alanine codon	This study
pALC2073Ω*sprG1*$_{312}$-*STOP*$_1$*-F10E-F13E/sprF1*	pALC2073Ω*sprG1*$_{312}$-*STOP*$_1$*/sprF1* with the two codons UUC, encoding to the two phenylalanine F10 and F13, mutated to the GAA glutamic acid codon	This study
pALC2073Ω*sprG1*$_{312}$-*STOP*$_1$ *1XFlag-Nt/sprF1*	pALC2073Ω*sprG1*$_{312}$-*STOP*$_1$*/sprF1* with a 1XFLAG sequence after the first AUG codon of SprG1$_{31}$ peptide	This study
pALC2073Ω*sprG1*$_{312}$-*STOP*$_1$ *1XFlag-Ct/sprF1*	pALC2073ΩsprG1$_{312}$-STOP$_1$/sprF1 with a 1XFLAG sequence before the termination codon of the internal coding sequence of SprG1$_{312}$	This study

Supplementary Table 3. DNA primers used in this study.

Genetic constructions		
DNA	***Sequences***	***Purposes***
SprG1$_{311}$**KpnI**_forw<br>SprG1**EcoRI**_rev	ATCG**GGTACC**CTTTGAGCAAGTTGGATAGATGGTGGCTAT<br>ATCG**GAATTC**GAAAAAGGGCAACATGCGCAAACAT	pALCΩ*sprG1*$_{312}$
SprG1311pALCKpnI_forw<br>SprG1SprF1EcoRI_rev	ATCG**GGTACC**CTTTGAGCAAGTTGGATAGATGGTGGCTAT<br>ATCG**GAATTC**AAAAGACGACTAATAAGCCGTCTAT	pALCΩ*sprG1*$_{312}$*/sprF1*
SprG1-**STOP1**_forw<br>SprG1-**STOP1**_rev	AAGGAGGTGGTGCCT**TAA**GTGGCATTACTGAAA<br>TTTCAGTAATGCCAC**TTA**AGGCACCACCTCCTT	pALCΩ*sprG1*$_{312}$-<br>*STOP*$_{1}$*/sprF1*
SprG1-**M14A**_forw<br>SprG1-**M14A**_rev	TAGAAAGGAGACGCCTA**GCC**ATTACAATTAGTACCATG<br>CATGGTACTAATTGTAAT**GGC**TAGGCGTCTCCTTTCTA	pALCΩ*sprG1*$_{312}$-<br>*M14A/sprF1*
SprG1-**STOP1,2,14**_forw<br><br>SprG1-**STOP1,2,14**_rev	AAGGAGGTGGTGCCT**TAATAA**GCATTACTGAAATCTTTAGA-<br>AGGAGACGCCTA**TAA**ATTACAATTAGTACCATG<br>CATGGTACTAATTGTAAT**TTA**TAGGCGTCTCCTTTCTAAAGAT<br>T-TCAGTAATGC**TTATTA**AGGCACCACCTCCTT	pALCΩ*sprG1*$_{312}$-<br>*STOP*$_{1,2,14}$*/sprF1*
SprG1-**STOP17**_forw<br>SprG1-**STOP17**_rev	GCCTATAAATTACA**TAA**AGTACCATGTTGC<br>GCAACATGGTACT**TTA**TGTAATTTATAGGC	pALCΩ*sprG1*$_{312}$-<br>*STOP*$_{1,2,14,17}$*/sprF1*
SprG1-*Flag3XCter*_forw<br><br><br>SprG1-*Flag3XCter*_rev	*GACTACAAAGACCATGACGGTGATTATAAAGATCATGACATCGAC*<br>*TACAAGGATGACGATGACAAG*TAACCATCGCTAACTTTGGCTG<br>GTTT<br>*CTTGTCATCGTCATCCTTGTAGTCGATGTCATGATCTTTATAATCA*<br>*CCGTCATGGTCTTTGTAGTC*TTTTTTATTGCTTAATTCAAT	pALCΩ*sprG1*$_{312}$-<br>*3XFlag-Ct/sprF1*<br>pALC2073Ω*sprG1*$_{312}$-<br>*3XFlag-Ct-*<br>*STOP*$_{1}$*/sprF1*<br>pALC2073Ω*sprG1*$_{312}$-<br>*3XFlag-Ct-*<br>*M14A/sprF1*<br>pALC2073Ω*sprG1*$_{312}$-<br>*3XFlag-Ct-*<br>*STOP*$_{1,2,14}$*/sprF1*<br>pALC2073Ω*sprG1*$_{312}$-<br>*3XFlag-Ct-*<br>*STOP*$_{1,2,14,17}$*/sprF1*

SprG1-**Δ9**_forw<br>SprG1-**Δ9**_rev	GGTCTAGTAATC**TAA**CTTATTGAATT<br>AATTCAATAAGTTAGA**TTA**CTAGACC	pALC2073Ω*sprG1*$_{312}$-*STOP*$_{1}$-*Δ9/sprF1*
SprG1-**Δ2**_forw<br>SprG1-**Δ2**_rev	GAATTAAGCAAT**TAA**AAATAACCATCGC<br>GCGATGGTTATTT**TTA**ATTGCTTAATTC	pALC2073Ω*sprG1*$_{312}$-*STOP*$_{1}$-*Δ2/sprF1*
SprG1-**F10A-F13A**_forw<br>SprG1-**F10A-F13A**_rev	GTTGCAG**GCA**GGTTTA**GCA**CTTATTG<br>CAATAAG**TGC**TAAACC**TGC**CTGCAAC	pALC2073Ω*sprG1*$_{312}$-*STOP*$_{1}$-*F10A-F13A/sprF1*
SprG1-**F10E-F10E**_forw<br>SprG1-**F10E-F10E**_rev	GTTGCAG**GAA**GGTTTA**GAA**CTTATTGC<br>GCAATAAG**TTC**TAAACC**TTC**CTGCAAC	pALC2073Ω*sprG1*$_{312}$-*STOP*$_{1}$-*F10E-F13E/sprF1*
SprG1-**K2K3**_forw<br>SprG1-**K2K3**_rev	GGAGACGCCTAATG**AAAAAA**ATTACAATTAG<br>CTAATTGTAAT**TTTTTT**CATTAGGCGTCTCC	pALC2073Ω*sprG1*$_{312}$-*STOP*$_{1}$-K_2K_3*/sprF1*
SprG1-*Flag1XNter*_forw<br>SprG1-*Flag1XNter*_rev	CGCCTAATG*GACTACAAAGACGATGACGACAAG*ATTACAATTAG<br>CTAATTGTAAT*CTTGTCGTCATCGTCTTTGTAGTC*CATTAGGCG	pALC2073Ω*sprG1*$_{312}$-*STOP*$_{1}$ *1XFlag-Nt/sprF1*
SprG1-*Flag1XCter*_forw<br>SprG1-*Flag1XCter*_rev	GCAATAAAAAA*GACTACAAAGACGATGACGACAAG*TAACCATCGC<br>GCGATGGTTA*CTTGTCGTCATCGTCTTTGTAGTC*TTTTTTATTGC	pALC2073Ω*sprG1*$_{312}$-*STOP*$_{1}$ *1XFlag-Ct/sprF1*

**Northern blot**		
DNA	***Sequences***	***Purposes***
SprG1-NB	CAGCCAAAGTTAGCGATGGT	Detection of *sprG1*$_{312}$
SprF1-NB	TAACTTTGGCTGGTTTCGATGGTT	Detection of SprF1
ARN5S - NB	CGTAAGTTCGACTACATCG	Detection of 5S rRNA
FLAG-NB	ATCCTTGTAGTCGATGTCATGATCTT	Detection of flagged RNAs

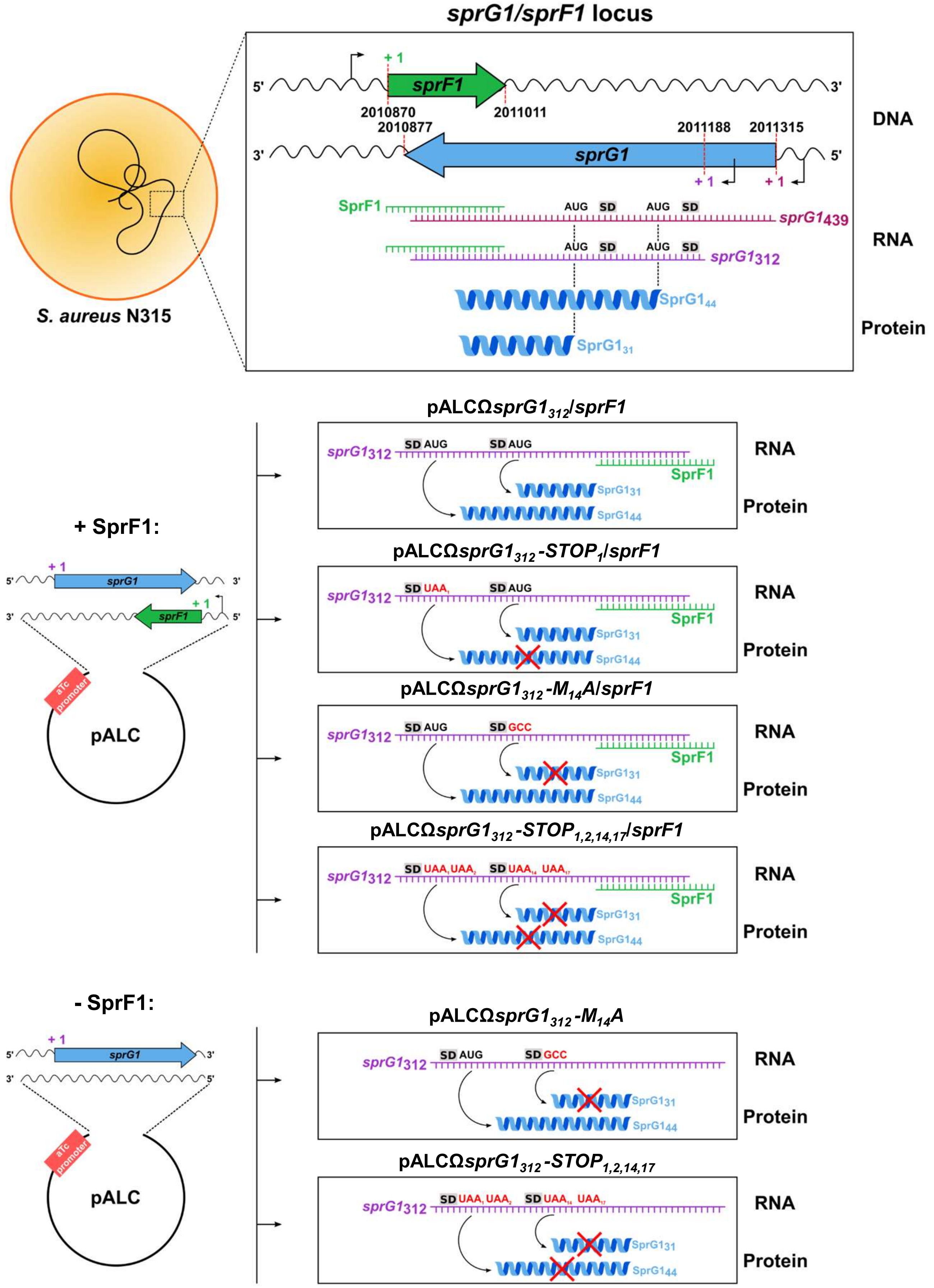

Supplementary Figure 1: Genetic map of the wild-type *sprG1*/*sprF1* locus and the various constructs in the pALC vector used in this work. The AUG54, GUG57 and AUG93 initiation codons and the AUU102 isoleucine codon were replaced with a UAA termination codon that refers to $STOP_1$, $STOP_2$, $STOP_{14}$ and $STOP_{17}$ mutations respectively. The AUG93 initiation codon was replaced with a GCC alanine codon that refers to M14A mutation.

CUUUGAGCAAGUUGGAUAGAUGGUGGCUAUCU
20

GAGUAUAAGGAGGUGGUGCCUAUGGUGGCAU
40 60
STOP$_1$ STOP$_2$

UACUGAAAUCUUUAGAAAGGAGACGCCUAAUG
80
M14A/STOP$_{14}$

AUUACAAUUAGUACCAUGUUGCAGUUUGGUUU
100 120
STOP$_{17}$

AUUCCUUAUUGCAUUGAUAGGUCUAGUAAUCA
140

AGCUUAUUGAAUUAAGCAAUAAAAAAUAACCA
160 180

UCGCUAACUUUGGCUGGUUUCGAUGGUUAAAU
200 220

GGUUAUUAAUUUAAUCUUUAAUCUAAAAUAGC
240

CACCGUCUUUUUAACGGGCUCACUAGGGUAAC
260 280

AUGUUUGCGCAUGUUGCCCUUUUUC
300

Supplementary Figure 2: The *sprG1$_{312}$* mRNA sequence. The open reading frame is highlighted in grey. The AUG54, GUG57 and AUG93 initiation codons and the AUU102 isoleucine codon were replaced with a UAA termination codon that refers to STOP$_1$, STOP$_2$, STOP$_{14}$ and STOP$_{17}$ mutations respectively. The AUG93 initiation codon was replaced with a GCC alanine codon that refers to M14A mutation.

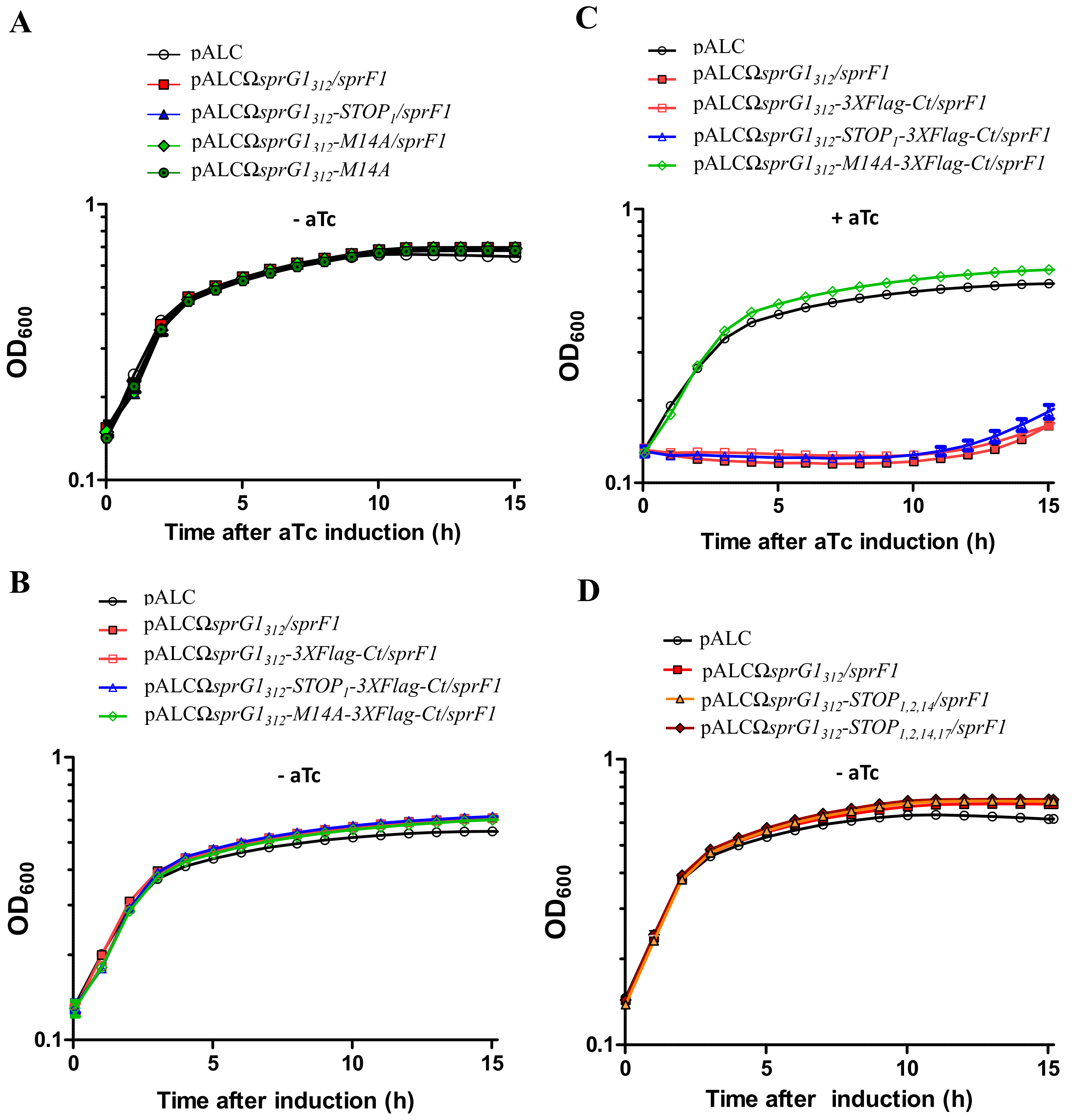

Supplementary Figure 3: Growth kinetics of *Staphylococcus aureus* strains. (A) Growth kinetics of *S. aureus* N315Δ*sprG1/sprF1* strains carrying pALC, pALCΩ*sprG1*$_{312}$*/sprF1,* pALCΩ*sprG1*$_{312}$*-STOP*$_{1}$*/sprF1,* pALCΩ*sprG1*$_{312}$*-M14A/sprF1* or pALCΩ*sprG1*$_{312}$*-M14A* cultivated in MH medium in the absence of aTc. (B) Growth kinetics of *S. aureus* N315Δ*sprG1/sprF1* strains carrying pALC, pALCΩ*sprG1*$_{312}$*/sprF1,* pALCΩ*sprG1*$_{312}$*-3XFlag-Ct/sprF1,* pALCΩ*sprG1*$_{312}$*-STOP*$_{1}$*-3XFlag-Ct /sprF1* or pALCΩ*sprG1*$_{312}$*-M14A-3XFlag-Ct/sprF1* cultivated in MH medium until the exponential growth phase and incubated in the presence of 0.25 µM aTc. (C) Growth kinetics of *S. aureus* N315Δ*sprG1/sprF1* strains carrying pALC, pALCΩ*sprG1*$_{312}$*/sprF1,* pALCΩ*sprG1*$_{312}$*-STOP*$_{1,2,14}$*/sprF1* or pALCΩ*sprG1*$_{312}$*-STOP*$_{1,2,14,17}$*/sprF1* cultivated in MH medium in the absence of aTc. (D) Growth kinetics of *S. aureus* N315Δ*sprG1/sprF1* strains carrying pALC, pALCΩ*sprG1*$_{312}$*/sprF1,* pALCΩ*sprG1*$_{312}$*-3XFlag-Ct/sprF1,* pALCΩ*sprG1*$_{312}$*-STOP*$_{1}$*-3XFlag-Ct/sprF1* or pALCΩ*sprG1*$_{312}$*-M14A-3XFlag-Ct/sprF1* cultivated in MH medium in the absence of aTc. Error bars show the means and standard deviations of three biological replicates (n=3).

A

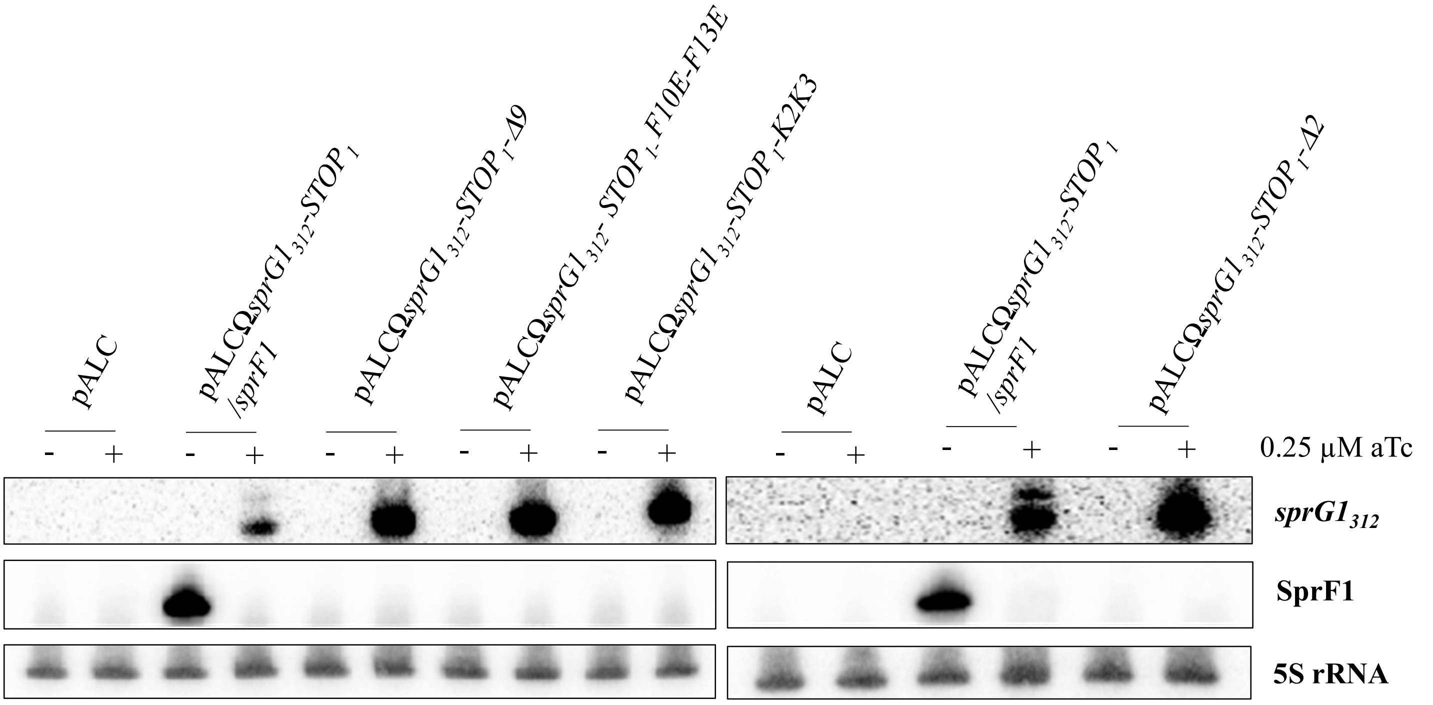

B

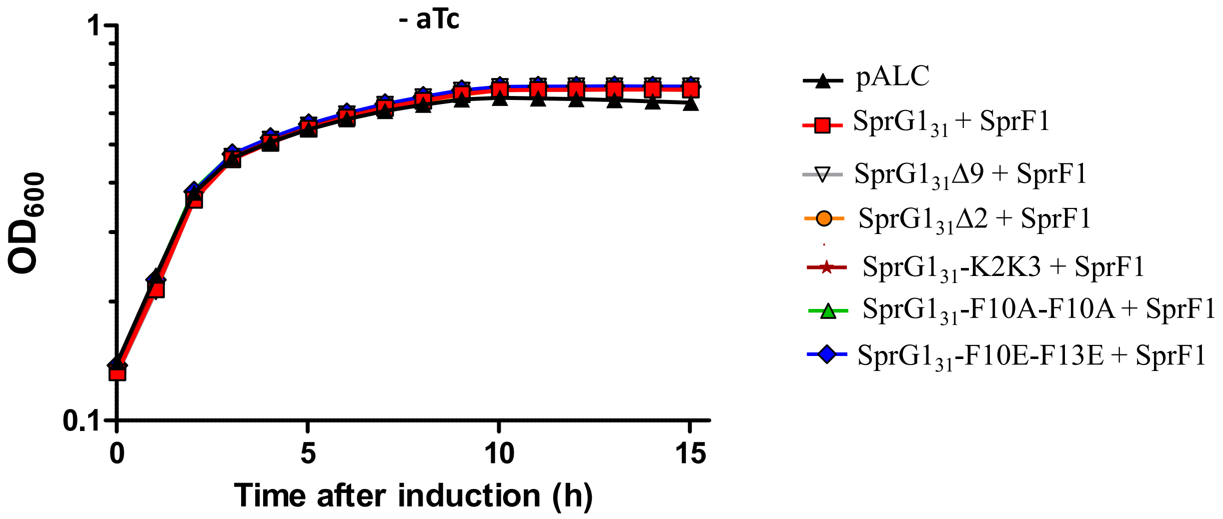

Supplementary Figure 4: Northern blot analysis of *sprG1$_{312}$* mRNA expression and growth kinetics in the absence of aTc for SprG1$_{31}$ mutants. (A) *S. aureus* N315Δ*sprG1/sprF1* strains carrying pALC, pALCΩ*sprG1$_{312}$-STOP$_1$/sprF1,* pALCΩ*sprG1$_{312}$-STOP$_1$-Δ9,* pALCΩ*sprG1$_{312}$-STOP$_1$-Δ2,* pALCΩ*sprG1$_{312}$-STOP$_1$-K2K3* or pALCΩ*sprG1$_{312}$-STOP$_1$-F10E-F13E* were cultivated in MH medium until exponential growth phase and incubated in the absence (-) or presence (+) of 0.25 µM aTc. After RNA extraction, northern blot analysis was done on *sprG1$_{312}$*, *sprG1$_{312}$* mutants and SprF1 expression with 5S rRNA used as the loading control. (B) Growth kinetics of *S. aureus* N315Δ*sprG1/sprF1* strains carrying pALC, pALCΩ*sprG1$_{312}$-STOP$_1$/sprF1* (SprG1$_{31}$ + SprF1), pALCΩ*sprG1$_{312}$-STOP$_1$-Δ9/sprF1* (SprG1$_{31}$Δ9 + SprF1), pALCΩ*sprG1$_{312}$-STOP$_1$-Δ2/sprF1* (SprG1$_{31}$Δ2 + SprF1), pALCΩ*sprG1$_{312}$-STOP$_1$-K2K3/sprF1* (SprG1$_{31}$-K2K3 + SprF1), pALCΩ*sprG1$_{312}$-STOP$_1$-F10A-F13A/sprF1* (SprG1$_{31}$-F10A-F13A + SprF1) or pALCΩ*sprG1$_{312}$-STOP$_1$-F10E-F13E/sprF1* (SprG1$_{31}$-F10E-F13E + SprF1) cultivated in MH medium in the absence of aTc. Error bars show the means and standard deviations of three biological replicates (n=3).

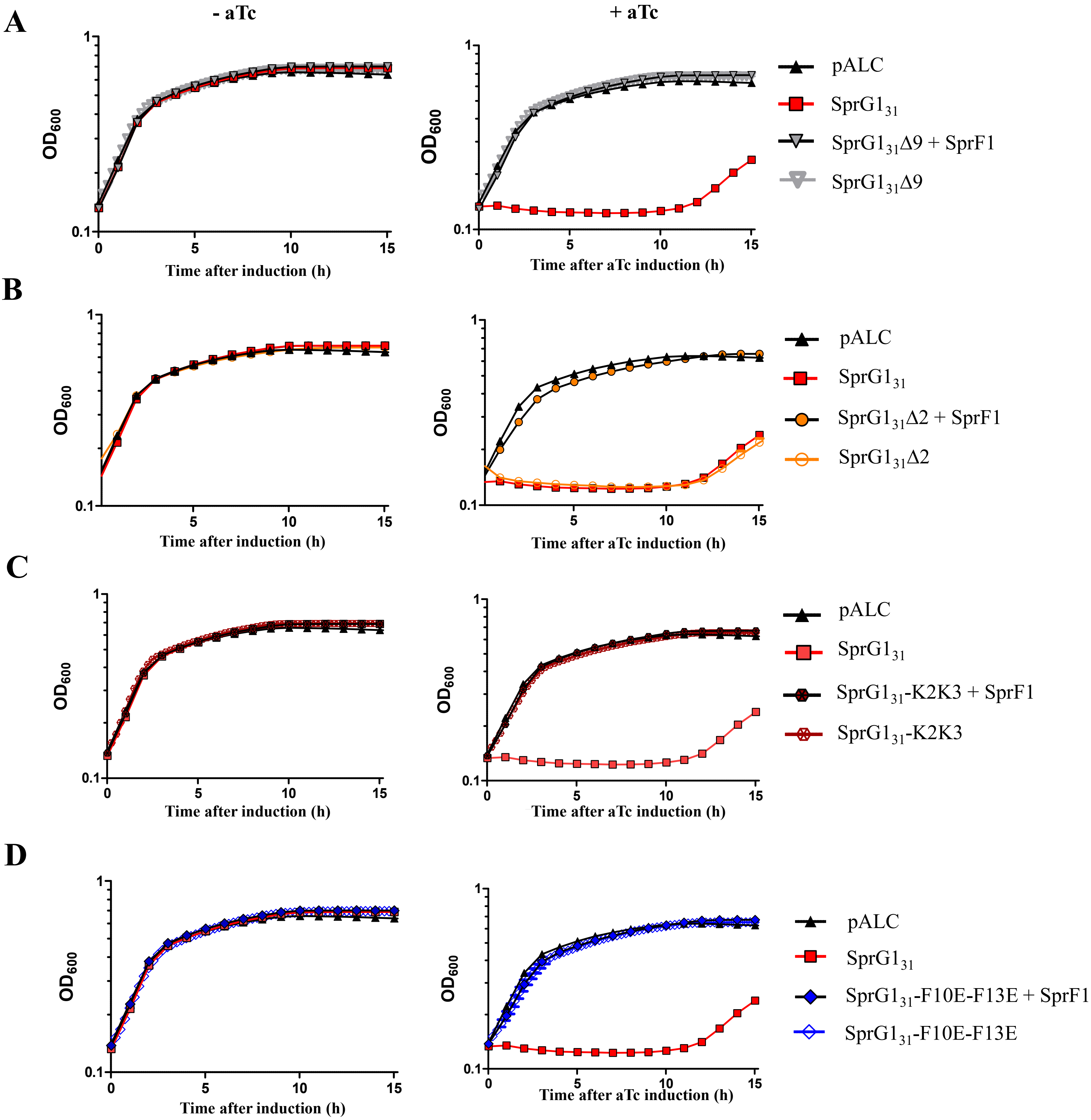

Supplementary Figure 5: Growth kinetics of SprG1$_{31}$ mutants in the absence or presence of SprF1 antitoxin. (A) Growth kinetics of *S. aureus* N315Δ*sprG1/sprF1* strains carrying pALC, pALCΩ*sprG1$_{312}$-STOP$_{1}$/sprF1* (SprG1$_{31}$), pALCΩ*sprG1$_{312}$-STOP$_{1}$-Δ9/sprF1* (SprG1$_{31}$Δ9 + SprF1) or pALCΩ*sprG1$_{312}$-STOP$_{1}$-Δ9* (SprG1$_{31}$Δ9) cultivated in MH medium in the absence (-) or presence (+) of 0.25 µM aTc. (B) Growth kinetics of *S. aureus* N315Δ*sprG1/sprF1* strains carrying pALC, pALCΩ*sprG1$_{312}$-STOP$_{1}$/sprF1* (SprG1$_{31}$), pALCΩ*sprG1$_{312}$-STOP$_{1}$-Δ2/sprF1* (SprG1$_{31}$Δ2 + SprF1) or pALCΩ*sprG1$_{312}$-STOP$_{1}$-Δ2* (SprG1$_{31}$Δ2) cultivated in MH medium in the absence (-) or presence (+) of 0.25 µM aTc. (C) Growth kinetics of *S. aureus* N315Δ*sprG1/sprF1* strains carrying pALC, pALCΩ*sprG1$_{312}$-STOP$_{1}$/sprF1* (SprG1$_{31}$), pALCΩ*sprG1$_{312}$-STOP$_{1}$-K2K3/sprF1* (SprG1$_{31}$-K2K3 + SprF1) or pALCΩ*sprG1$_{312}$-STOP$_{1}$-K2K3* (SprG1$_{31}$-K2K3) cultivated in MH medium in the absence (-) or presence (+) of 0.25 µM aTc. (D) Growth kinetics of *S. aureus* N315Δ*sprG1/sprF1* strains carrying pALC, pALCΩ*sprG1$_{312}$-STOP$_{1}$/sprF1* (SprG1$_{31}$), pALCΩ*sprG1$_{312}$-STOP$_{1}$-F10E-F13E/sprF1* (SprG1$_{31}$-F10E-F13E + SprF1) or pALCΩ*sprG1$_{312}$-STOP$_{1}$-F10E-F13E* (SprG1$_{31}$-F10E-F13E) cultivated in MH medium in the absence (-) or presence (+) of 0.25 µM aTc. Error bars show the means and standard deviations of three biological replicates (n=3).

A

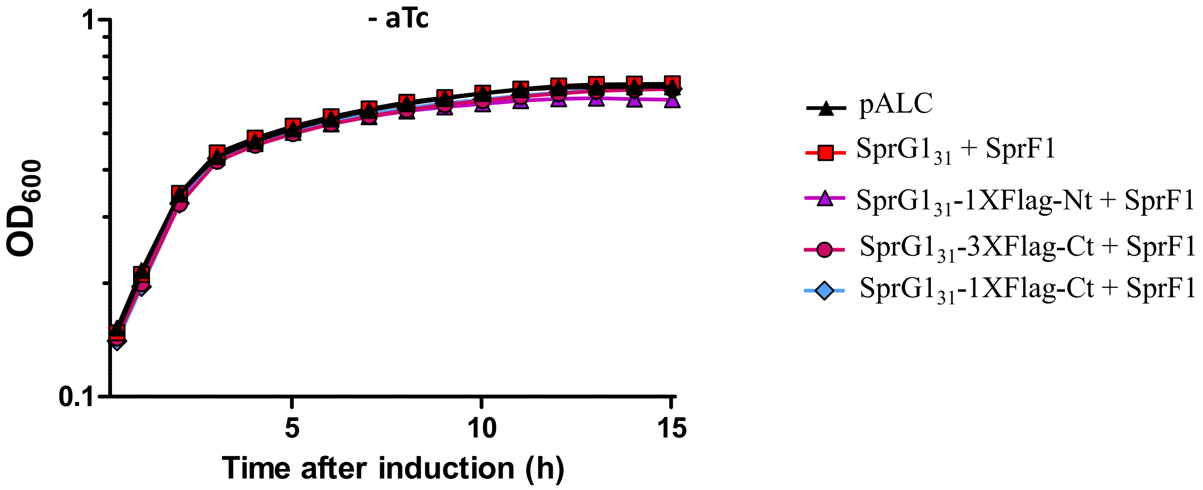

B

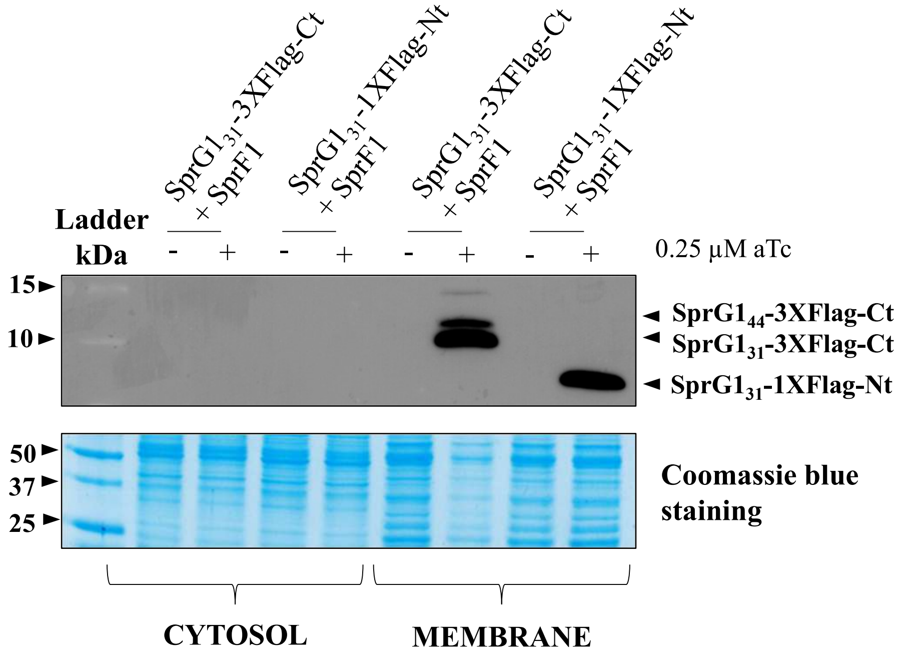

Supplementary Figure 6: Growth kinetics and western blot analysis of flagged SprG1$_{31}$ mutants. (A) Growth kinetics of *S. aureus* N315Δ*sprG1/sprF1* strains carrying pALC, pALCΩ*sprG1$_{312}$-STOP$_{1}$/sprF1* (SprG1$_{31}$ + SprF1), pALCΩ*sprG1$_{312}$-STOP$_{1}$-1XFlag-Nt/sprF1* (SprG1$_{31}$-1XFlag-Nt + SprF1), pALCΩ*sprG1$_{312}$-STOP$_{1}$-1XFlag-Ct/sprF1* (SprG1$_{31}$-1XFlag-Ct + SprF1) or pALCΩ*sprG1$_{312}$-STOP$_{1}$-3XFlag-Ct-/sprF1* (SprG1$_{31}$-3XFlag-Ct + SprF1) cultivated in MH medium in the absence of aTc. Error bars show the means and standard deviations of three biological replicates (n=3). (B) *S. aureus* N315Δ*sprG1/sprF1* strains carrying pALCΩ*sprG1$_{312}$-3XFlag-Ct-STOP$_{1}$/sprF1* (SprG1$_{31}$-3XFlag-Ct + SprF1) or pALCΩ*sprG1$_{312}$-STOP$_{1}$-1XFlag-Nt/sprF1* (SprG1$_{31}$-1XFlag-Nt + SprF1) were cultivated in MH medium until exponential growth phase and incubated in the absence (-) or presence (+) of 0.25 µM aTc. After cell fractionation, the expression of the *sprG1$_{312}$*-encoded flagged peptides, SprG1$_{44}$-3XFlag-Ct, SprG1$_{31}$-3XFlag-Ct and SprG1$_{31}$-1XFlag-Nt, was analyzed by western blot by using anti-FLAG antibodies. Coomassie blue staining was used as the loading control.

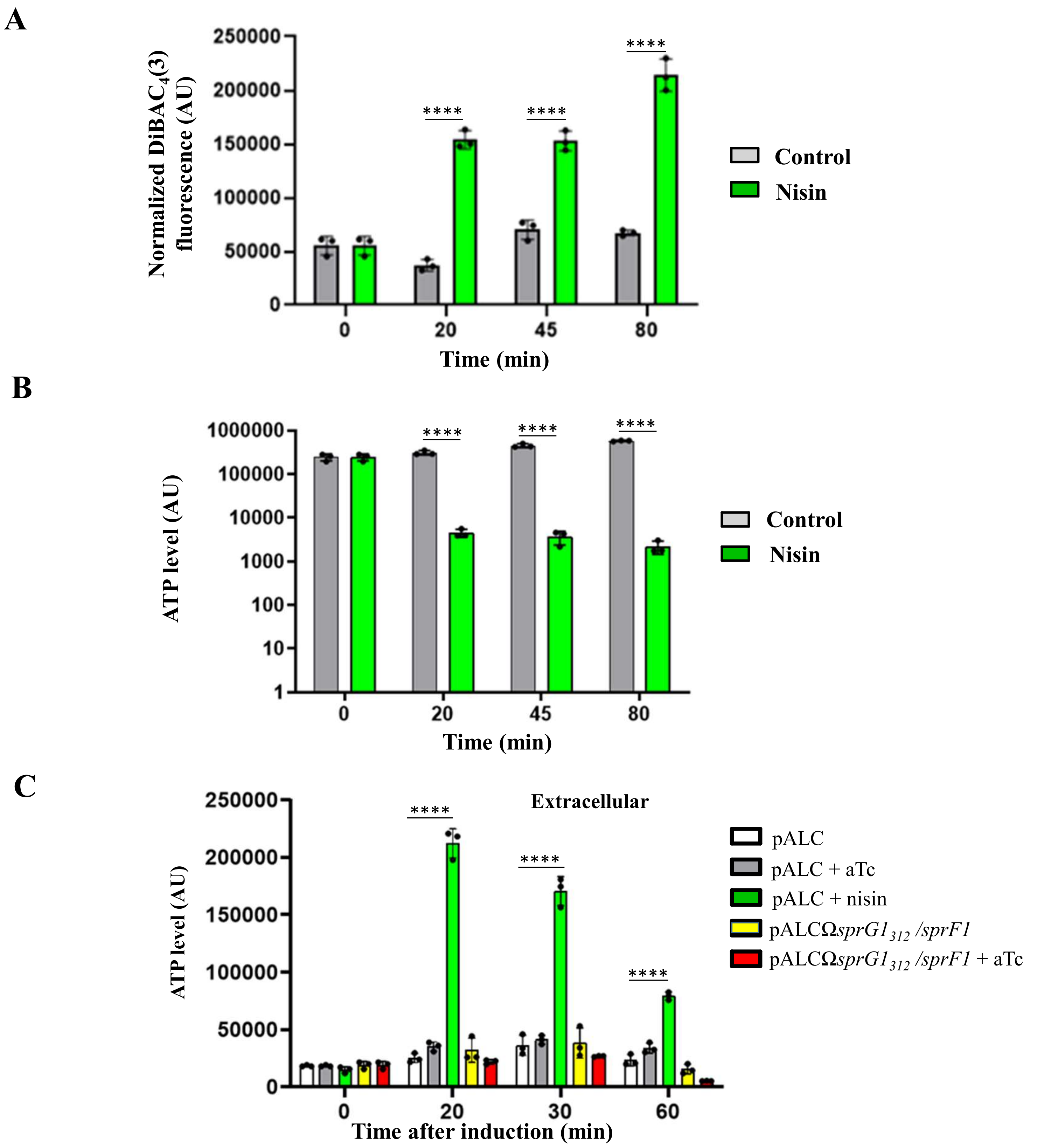

Supplementary Figure 7: The pore-forming peptide nisin causes *Staphylococcus aureus* membrane depolarization followed by an intracellular ATP drop and an increase of ATP leakage into the extracellular medium. (A-B) *S. aureus* N315Δ*sprG1/sprF1* strains carrying pALC was cultivated in MH medium until the exponential growth phase and incubated in absence (Control) or presence of 12.5 µg/mL nisin. (A) At each time point, bacteria were resuspended in PBS and stained with 0.5 µg/mL $DiBAC_4(3)$. $DiBAC_4(3)$ fluorescence was measured in a microplate reader and normalized with OD_{600}. (B) At each time point, bacteria were resuspended in MH and incubated with BacTiter-Glo Microbial Cell Viability Assay. Luminescence values were normalized with OD_{600}. (C) *S. aureus* N315Δ*sprG1/sprF1* strains carrying pALC and pALCΩ$sprG1_{312}$/*sprF1* were cultivated in MH medium until the exponential growth phase and incubated in absence or presence of 0.25 µM aTc or 12.5 µg/mL nisin used as the positive control. At each time point, supernatants (extracellular fraction) were incubated with BacTiter-Glo Microbial Cell Viability Assay. Luminescence values normalized with OD_{600}. Error bars show the means and standard deviations of three biological replicates (n=3). Statistical significance was calculated with the two-way ANOVA with Tukey's correction. ****P<0.0001. AU refers to arbitrary unit.

A

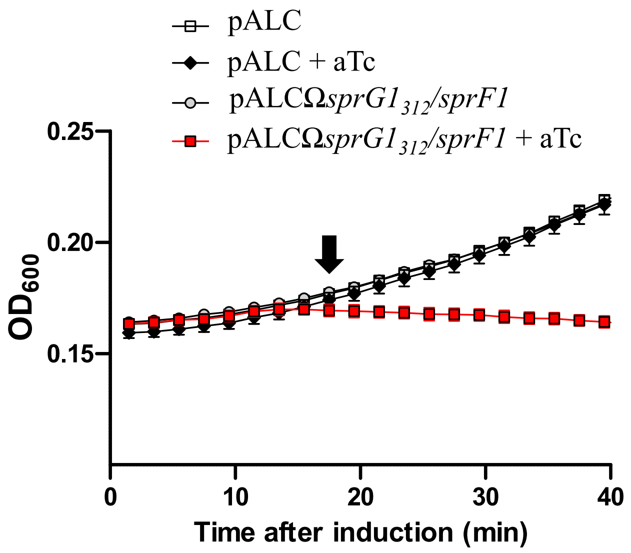

B

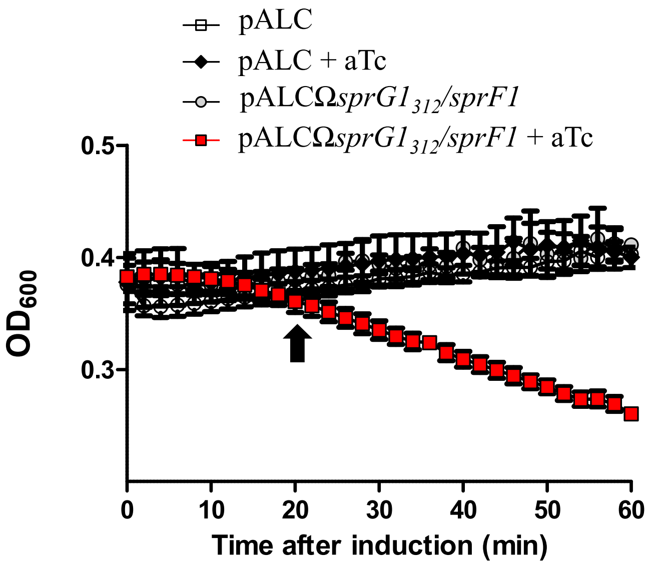

Supplementary Figure 8: SprG1$_{44}$ and SprG1$_{31}$ arrest the growth of *Staphylococcus aureus* within 20 minutes after overexpression. (A) *S. aureus* N315Δ*sprG1/sprF1* strains carrying pALC and pALCΩ*sprG1$_{312}$/sprF1* were cultivated in MH medium until the exponential growth phase and incubated in absence or presence of 0.25 µM aTc. (B) *S. aureus* N315Δ*sprG1/sprF1* strains carrying pALC or pALCΩ*sprG1$_{312}$/sprF1* were cultivated in MH medium until the exponential growth phase, resuspended in PBS supplemented with 25 mM glucose and incubated in absence or presence of 0.25 µM aTc. (A-B) OD_{600} was measured in a microplate reader. The results are expressed as the mean ± SD of three biological replicates (n=3). Arrows indicate the time for which the *S. aureus* growth is inhibited by the overexpression of SprG1$_{44}$ and SprG1$_{31}$.

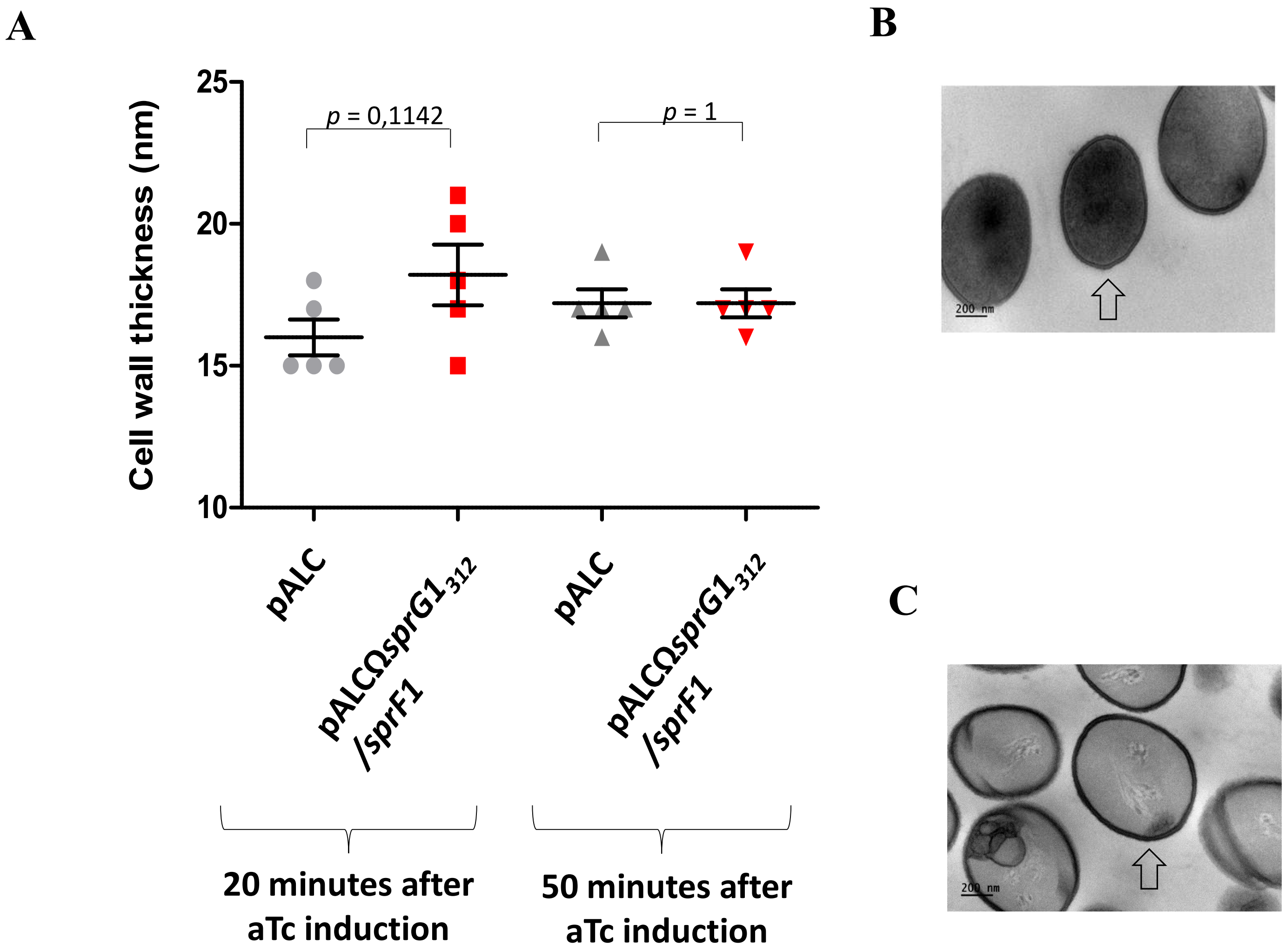

Supplementary Figure 9: Measurement of cell wall thickness by transmission electron microscopy. *S. aureus* N315Δ*sprG1/sprF1* strains carrying pALC or pALCΩ*sprG1*$_{312}$*/sprF1* were cultivated in MH medium until the exponential growth phase and incubated with 0.25 µM aTc during 20 or 50 min. (A) Cell wall thickness of *S. aureus* was measured using transmission electron microscopy. The results are expressed as the mean ± SD of 5 representative bacteria. *P* values inferred using the Student's *t*-test. (B-C) Captions of representative bacteria (arrow) carrying pALC (B) or pALCΩ*sprG1*$_{312}$*/sprF1* (C) 20 minutes after aTc induction.

Supplemental references

Bateman, B. T., Donegan, N. P., Jarry, T. M., Palma, M. & Cheung, A. L. (2001). Evaluation of a Tetracycline-Inducible Promoter in *Staphylococcus aureus* In Vitro and In Vivo and Its Application in Demonstrating the Role of *sigB* in Microcolony Formation. (E.I. Tuomanen, Hrsg.)*Infection and Immunity*, *69* (12), 7851–7857. doi:10.1128/IAI.69.12.7851-7857.2001

Kreiswirth, B. N., Löfdahl, S., Betley, M. J., O'Reilly, M., Schlievert, P. M., Bergdoll, M. S. et al. (1983). The toxic shock syndrome exotoxin structural gene is not detectably transmitted by a prophage. *Nature*, *305* (5936), 709–712. doi:10.1038/305709a0

Okonogi, K., Noji, Y., Kondo, M., Imada, A. & Yokota, T. (1989). Emergence of methicillin-resistant clones from cephamycin-resistant *Staphylococcus aureus*. *Journal of Antimicrobial Chemotherapy*, *24* (5), 637–645. doi:10.1093/jac/24.5.637

Pinel-Marie, M.-L., Brielle, R. & Felden, B. (2014). Dual Toxic-Peptide-Coding Staphylococcus aureus RNA under Antisense Regulation Targets Host Cells and Bacterial Rivals Unequally. *Cell Reports*, *7* (2), 424–435. doi:10.1016/j.celrep.2014.03.012
